# Milk polar lipids favorably alter circulating and intestinal ceramide and sphingomyelin species in postmenopausal women

**DOI:** 10.1172/jci.insight.146161

**Published:** 2021-05-24

**Authors:** Mélanie Le Barz, Cécile Vors, Emmanuel Combe, Laurie Joumard-Cubizolles, Manon Lecomte, Florent Joffre, Michèle Trauchessec, Sandra Pesenti, Emmanuelle Loizon, Anne-Esther Breyton, Emmanuelle Meugnier, Karène Bertrand, Jocelyne Drai, Chloé Robert, Annie Durand, Charlotte Cuerq, Patrice Gaborit, Nadine Leconte, Annick Bernalier-Donadille, Eddy Cotte, Martine Laville, Stéphanie Lambert-Porcheron, Lemlih Ouchchane, Hubert Vidal, Corinne Malpuech-Brugère, David Cheillan, Marie-Caroline Michalski

**Affiliations:** 1 Univ Lyon, CarMeN laboratory, INSERM, INRAE, INSA Lyon, Université Claude Bernard Lyon 1, Charles Mérieux Medical School, 69310, Pierre-Bénite, France.; 2 TCentre de Recherche en Nutrition Humaine Rhône-Alpes, Univ-Lyon, CarMeN Laboratory, Université Claude Bernard Lyon1, Hospices Civils de Lyon, CENS, FCRIN/FORCE Network, 69310, Pierre-Bénite, France.; 3 Université Clermont Auvergne, INRAE, UNH, Unité de Nutrition Humaine, CRNH Auvergne, 63000, Clermont-Ferrand, France.; 4 ITERG, ZA Pessac-Canéjan, 11 Rue Gaspard Monge, 33610, Canéjan, France.; 5 Hospices Civils de Lyon, 69000, Lyon, France.; 6 Unité Maladies Héréditaires du Métabolisme, Service de Biochimie et Biologie Moléculaire Grand Est, Centre de Biologie et de Pathologie Est, Hospices Civils de Lyon, 69677, Bron, France.; 7 Unité de Nutrition Endocrinologie Métabolisme, Service de Biochimie, Centre de Biologie et de Pathologie Sud, Hospices Civils de Lyon, 69495, Pierre-Bénite, France.; 8 ACTALIA Dairy Products and Technologies, Avenue François Mitterrand, BP49, 17700, Surgères, France.; 9 ENILIA ENSMIC, Avenue François Mitterrand, 17700, Surgères, France.; 10 INRAE, Institut Agro, STLO (Science et Technologie du Lait et de l’Œuf), 35042, Rennes, France.; 11 Université Clermont Auvergne, INRAE, UMR 454, MEDIS, 63000, Clermont-Ferrand, France.; 12 Hospices Civils de Lyon, Centre Hospitalier Lyon-Sud, Service de chirurgie digestive, 69310, Pierre-Bénite, France.; 13 Université Claude Bernard Lyon 1, Faculté de médecine Lyon-Sud-Charles Mérieux, EMR 3738, 69600, Oullins, France.; 14 Université Clermont Auvergne, CNRS, SIGMA Clermont, Institut Pascal, 63000, Clermont-Ferrand, France.; 15 CHU Clermont-Ferrand, Unité de Biostatistique-Informatique Médicale, 63000, Clermont-Ferrand, France.

**Keywords:** Clinical Trials, Metabolism, Cardiovascular disease, Cholesterol, Lipid rafts

## Abstract

**BACKGROUND:**

High circulating levels of ceramides (Cer) and sphingomyelins (SM) are associated with cardiometabolic diseases. The consumption of whole fat dairy products, naturally containing such polar lipids (PL), is associated with health benefits, but the impact on sphingolipidome remains unknown.

**METHODS:**

In a 4-week randomized controlled trial, 58 postmenopausal women daily consumed milk PL-enriched cream cheese (0, 3, or 5 g of milk PL). Postprandial metabolic explorations were performed before and after supplementation. Analyses included SM and Cer species in serum, chylomicrons, and feces. The ileal contents of 4 ileostomy patients were also explored after acute milk PL intake.

**RESULTS:**

Milk PL decreased serum atherogenic C24:1 Cer, C16:1 SM, and C18:1 SM species (*P*_group_ < 0.05). Changes in serum C16+18 SM species were positively correlated with the reduction of cholesterol (*r* = 0.706), LDL-C (*r* = 0.666), and ApoB (*r* = 0.705) (*P* < 0.001). Milk PL decreased chylomicron content in total SM and C24:1 Cer (*P*_group_ < 0.001), parallel to a marked increase in total Cer in feces (*P*_group_ < 0.001). Milk PL modulated some specific SM and Cer species in both ileal efflux and feces, suggesting differential absorption and metabolization processes in the gut.

**CONCLUSION:**

Milk PL supplementation decreased atherogenic SM and Cer species associated with the improvement of cardiovascular risk markers. Our findings bring insights on sphingolipid metabolism in the gut, especially Cer, as signaling molecules potentially participating in the beneficial effects of milk PL.

**TRIAL REGISTRATION:**

ClinicalTrials.gov, NCT02099032, NCT02146339.

**FUNDING:**

ANR-11-ALID-007-01; PHRCI-2014: VALOBAB, no. 14-007; CNIEL; GLN 2018-11-07; HCL (sponsor).

## Graphical abstract

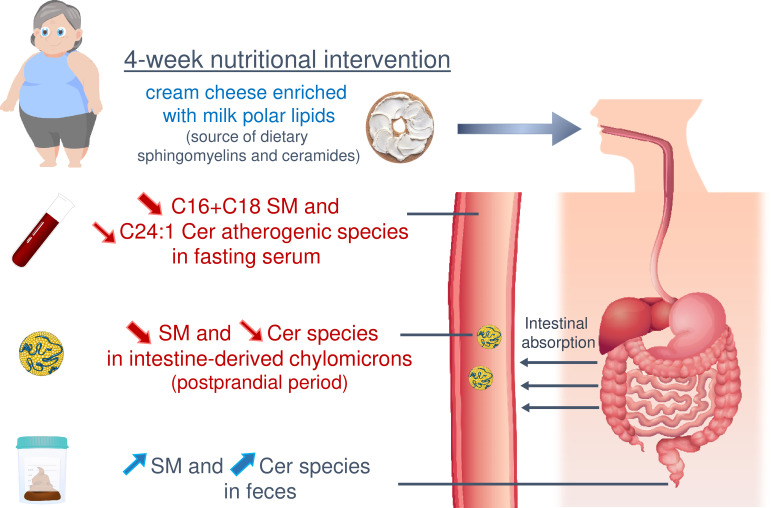

## Introduction

Sphingolipids (SP) represent a large class of bioactive polar lipids (PL) that play a pivotal role in structural and metabolic functions in the regulation of cardiometabolic homeostasis, intestinal health, and inflammatory signaling pathways (1–3). SP metabolism represents a vast and complex network (4), including a plethora of metabolically relevant species regulated by several key enzymes and metabolite fluxes in mammals (5). Dysregulated SP metabolism is related to negative health outcomes (6–8), and an increase in circulating ceramide (Cer) species (particularly C24:1 Cer), which are key precursors of the biosynthesis of several other SP molecules, correlates with markers of cardiometabolic complications (9–12). Sphingomyelins (SM) represent one of the most abundant SP families. High serum SM concentrations correlate with coronary heart diseases in obese patients (13), and high-fat diet increases specifically C16+18 SM species (i.e., C16:0, C16:1, C18:0, and C18:1 species) in rodents (14).

There is growing evidence showing that several factors may affect circulating SM and Cer concentrations such as drugs and lifestyle modifications, including exercise and dietary changes (15, 16). SP is found in plant and animal cell membranes; the daily SP intake represents approximately 0.3–0.4 g/day in humans (17), but the impact of such consumption on the endogenous sphingolipidome remains largely unknown. Cow milk has recently attracted more attention because it naturally contains SM and Cer (SM accounting for ~25% of PL in the milk fat globule membrane [MFGM]), including C20+22 species (i.e., C20:0, C20:1, C22:0, and C22:1 species) that are found in higher amounts in milk fat than in human blood. Recent metaanalysis and research papers highlight the beneficial cardiometabolic effects of the consumption of whole fat dairy product, which contain sizable amounts of milk PL (18, 19). Given that milk PL also contain C16+18 SM and C24:1 Cer species with potential deleterious effects, understanding the impact of milk SP on circulating levels and endogenous SP metabolism is of great importance.

**Figure 1:**
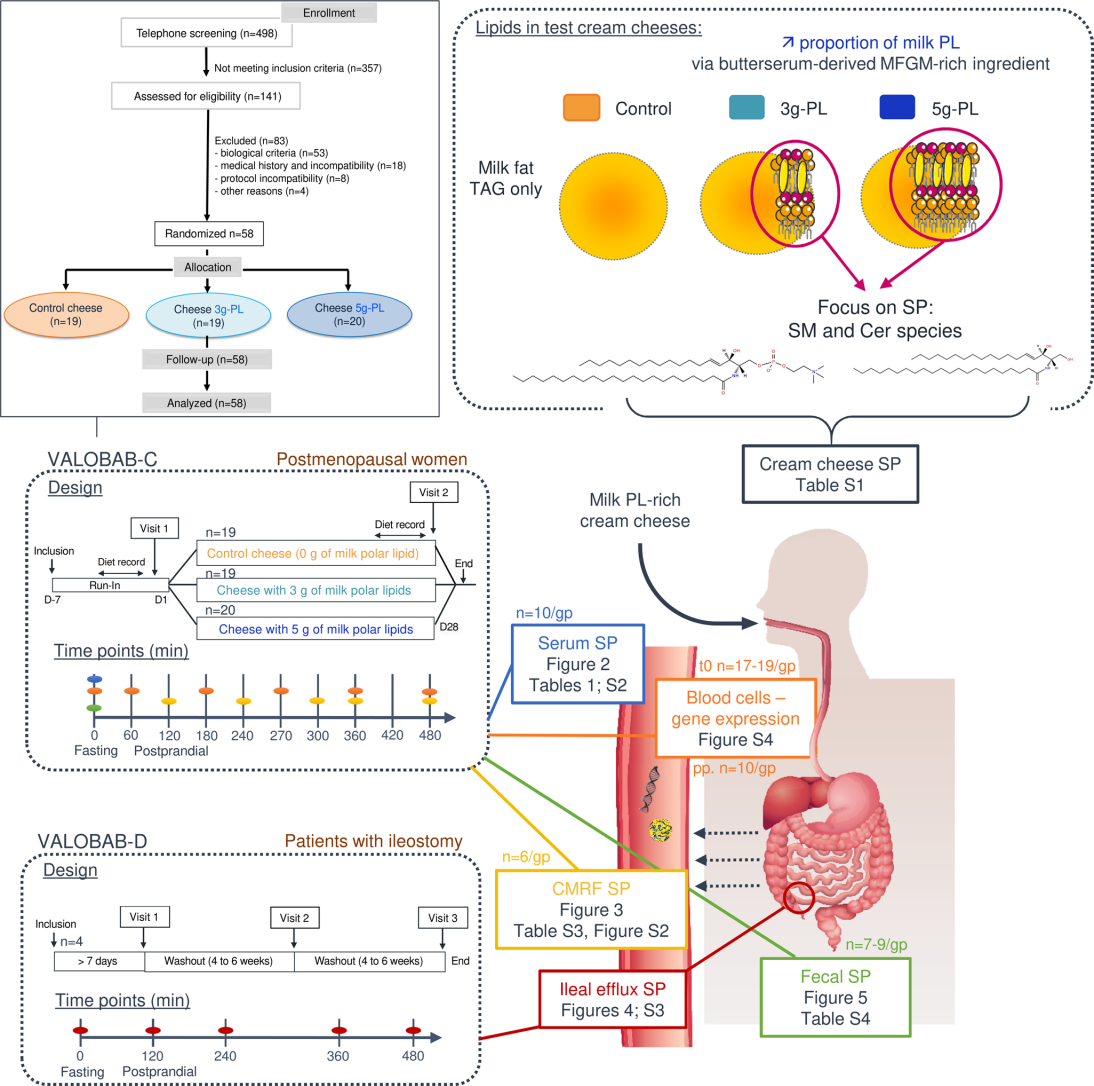
Design of VALOBAB-C and VALOBAB-D clinical trials and graphical summary of analyses performed on predefined secondary outcomes. In the VALOBAB-C clinical trial, 58 postmenopausal women were supplemented with test cream cheese containing either 0, 3, or 5 g of milk PL during 4 weeks. In the VALOBAB-D trial, 4 ileostomy patients were subjected to the acute consumption of the 3 test cheeses following a crossover study design. In both trials, during the exploratory visit, overnight fasted volunteers received a standardized breakfast rich in fat and sugars at time 0 and a meal containing the test cream cheese at time 240 minutes of the postprandial period. Tables and figures reporting specific results are listed. Cer, ceramides; CMRF, chylomicron-rich fraction; MFGM, milk fat globule membrane; PL, polar lipids; SP, sphingolipids; SM, sphingomyelins; TAG, triacylglycerols. Molecular structures were drawn using the LIPIDMAPS tool.

Preclinical studies revealed that milk SM supplementation prevents hyperlipemia, hypercholesterolemia, and low-grade inflammation and that it improves intestinal health related to gut microbiota modulations (2, 20–26). Buttermilk, a by-product of butter industry, represents a natural source of the SM-rich MFGM (1–10 g of milk PL/L) (27, 28). In the VALOBAB-C trial, we demonstrated recently that the 4-week consumption of milk PL–enriched test cheese decreases circulating total cholesterol (C) (primary outcome), triacylglycerols (TAG), LDL-C, and ApoB, at both fasting and postprandial states (Supplemental Figure 1; supplemental material available online with this article; https://doi.org/10.1172/jci.insight.146161DS1) in postmenopausal women at risk of cardiovascular disease (CVD) (29). However, there are still open questions regarding the potential involvement of the endogenous metabolism and intestinal fate of milk SP species in these benefits. We thus explored the prespecified secondary outcomes of the VALOBAB clinical trial by analyzing SM and Cer molecular species of particular interest in several biological compartments. We aimed to determine how the 4-week supplementation with milk PL impacts the circulating and fecal SM and Cer species at the fasting state, as well as their amount in intestine-derived chylomicrons during the postprandial period, in postmenopausal women at cardiovascular risk (Figure 1). We then verified whether this contributes to the beneficial effects of milk PL on lipid cardiovascular risk markers. In a complementary study conducted in ileostomy patients, we further aimed to identify the digestive fate of milk SP in the upper gastrointestinal tract after acute consumption of milk PL–rich meals (Figure 1).

## Results

### Sphingolipidome of serum SM and Cer molecular species is modified by milk PL.

The dietary intervention with milk PL significantly modified the amount of several molecular SP species in fasting serum. In the 5 g–PL group, the serum concentrations of the following species decreased between the first (V1) and second (V2) exploration visit: C16:1 SM (ΔV2–V1 CTL: +0.44 ± 0.97 μM; 3 g–PL: –0.64 ± 0.84 μM; 5 g–PL: –3.35 ± 0.50 μM; *P*_group_ = 0.007; post hoc analysis: *P*_CTL-5gPL_ = 0.006), C18:1 SM (ΔV2–V1 CTL: +1.02 ± 0.87 μM; 3 g–PL: –2.21 ± 0.69 μM; 5 g–PL: –2.99 ± 0.79 μM; *P*_group_ = 0.003; *P*_CTL-5gPL_ = 0.003), and C20:1 SM (ΔV2–V1 CTL: +0.62 ± 0.61 μM; 3 g–PL: –0.71 ± 0.50 μM; 5 g–PL: –1.57 ± 0.48 μM; *P*_group_ = 0.025; *P*_CTL-5gPL_ = 0.019) (Table 1). A decrease in serum C24:1 Cer species was also observed in milk PL groups regardless of dose (ΔV2–V1 CTL: +0.11 ± 0.08 μM; 3 g–PL: –0.19 ± 0.08 μM; 5 g–PL: –0.37 ± 0.18 μM; *P*_PL_=0.016), without any effect on the other identified Cer species. No difference between groups was observed in the circulating fasting concentrations of total SM and Cer and of phospholipids (Table 1). Parallel to their amount, changes in the relative abundance of fasting SM and Cer species after intervention — i.e., the proportion of each SM or Cer species in total analyzed serum SM or Cer, respectively — revealed decreased proportions of C18:1 SM species (*P*_group_ = 0.002) and C24:1 Cer species (ΔV2–V1 CTL: +0.65% ± 0.76%; 3 g–PL: –2.67% ± 0.96%; 5 g–PL: –2.65% ± 0.71%; *P*_group_ = 0.010; *P*_PL_ = 0.002; *P*_CTL-5g_ = 0.021; *P*_CTL-3g_ = 0.020; Supplemental Table 2). These beneficial effects were associated with the increase of the relative proportions of specific SM and Cer species usually poorly represented in human blood: C20:0 SM (ΔV2–V1 CTL: +0.14% ± 0.14%; 3 g–PL: +0.62% ± 0.08%; 5 g–PL: +0.95% ± 0.09%; *P*_group_ = 0.00005; *P*_CTL-5g_ = 0.00003; *P*_CTL-3g_ = 0.010), C22:1 SM (ΔV2–V1 CTL: –0.02% ± 0.10%; 3 g–PL: +0.65% ± 0.27%; 5 g–PL: +0.56% ± 0.23%; *P*_group_ = 0.07; *P*_PL_ = 0.021), and C20:0 Cer species (ΔV2–V1 CTL: –1.44% ± 0.65%; 3 g–PL: +0.38% ± 0.56%; 5 g–PL: +0.47% ± 0.60%; *P*_group_ = 0.057; *P*_PL_ = 0.016) (Supplemental Table 2).

### Milk PL–induced modulations of serum SM and Cer profiles are correlated with the decrease of CVD risk markers.

**Figure 2:**
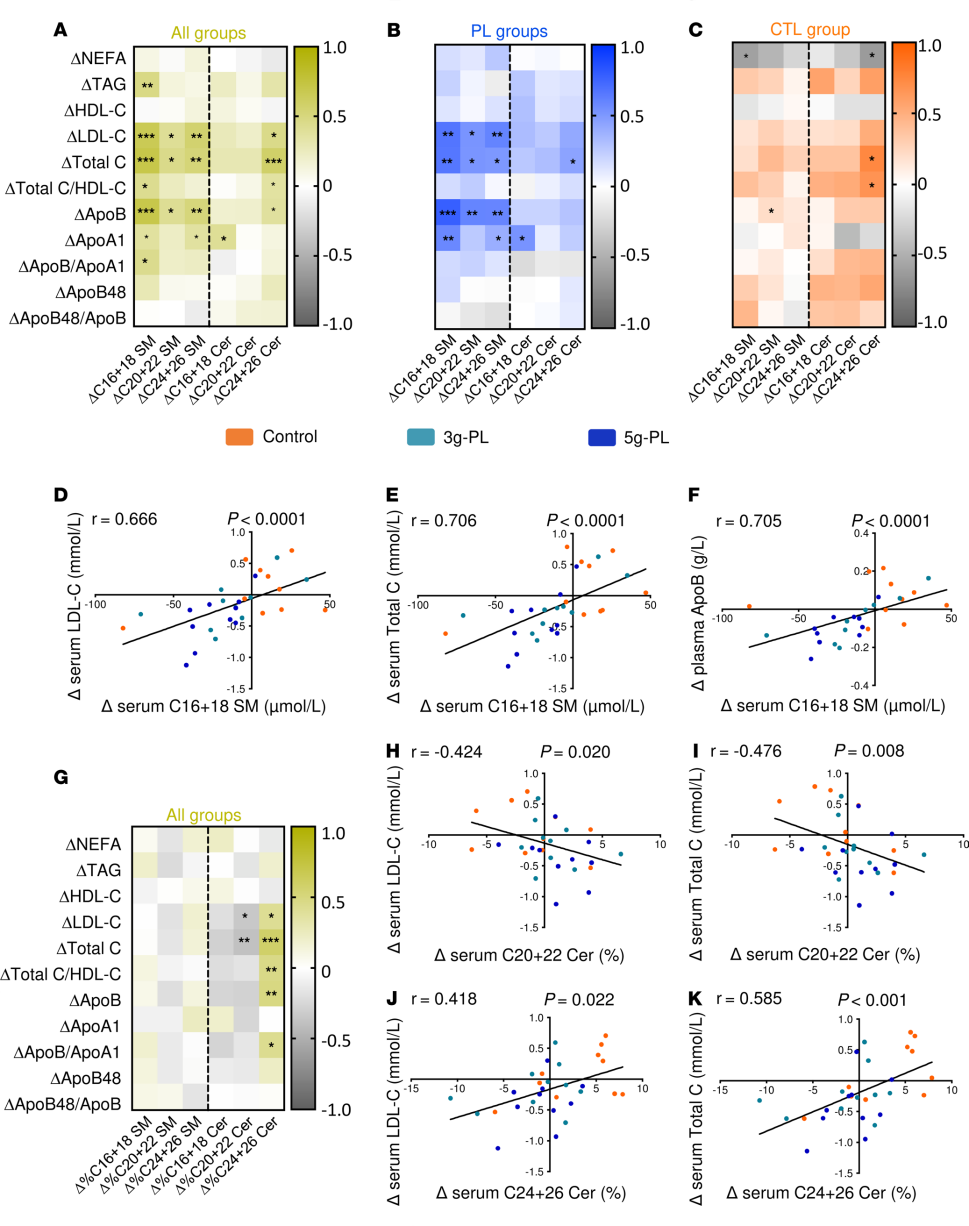
Major correlations between the impacts of milk PL supplementation on blood lipids and on serum SM and Cer (VALOBAB-C). (**A–C**, and **G**) Spearman’s correlations between blood lipids and serum SM and Cer species. All data are expressed as ΔV2–V1 at fasting. Yellow, all groups were considered for the analysis (**A** and **G**) (*n* = 30); blue, the 2 groups supplemented with either 3 or 5 g–milk PL only (*n* = 20); and orange, the control group only (*n* = 10). For panels **A**–**C**, and **G**, asterisks in bold represent correlations that remain significant after adjustment for clinical center, quartiles of volunteer age, and waist circumference. (**D**–**F** and **H**–**K**) Graphs illustrating specific Spearman’s correlations between the intervention impact on C16+18 SM species and on LDL-C (**D**), total C (**E**), and ApoB48 (**F**); between C20+22 Cer species proportions (%) and LDL-C (**H**), and total C (**I**); between C24-26 Cer species proportions (%) and LDL-C (**J**), and total C (**K**). Apo, apolipoprotein; C, cholesterol; Cer, ceramides; CTL, control; HDL, high density lipoprotein; LDL, low density lipoprotein; NEFA, nonesterified fatty acids; PL, polar lipids; SM, sphingomyelin; TAG, triacylglycerols.

Results demonstrated a significant correlation between change in serum SM (particularly C16+18 SM species) and ΔLDL-C, Δtotal C, and ΔApoB (Figure 2A, P** < 0.001). These correlations were mainly mediated by the dietary intervention regardless of milk PL dose (Figure 2B) versus no correlation in control group (Figure 2C), as also illustrated in Figure 2, D–F, by the specific correlations between ΔC16+18 SM species and ΔLDL-C (*r* = 0.666, *P* < 0.0001), Δtotal C (*r* = 0.706, *P* < 0.0001), and ΔApoB (*r* = 0.705, *P* < 0.0001). Fewer correlations were observed between changes in Cer concentrations and those of blood lipids. Because results revealed changes in the SP species proportions (Supplemental Table 2), we analyzed potential correlations with blood lipid concentrations (Figure 2G). Changes in C24+26 Cer species proportions (Δ%C24+26 Cer) positively correlated with ΔLDL-C (*r* = 0.418, *P* = 0.022), Δtotal C (*r* = 0.585, *P* < 0.001) (Figure 2, G, J, and K), and ΔApoB (*r* = 0.492, *P* = 0.006). Conversely, variations in C20+22 Cer species (Δ%C20+22 Cer) proportions negatively correlated with ΔLDL-C (*r* = –0.424, *P* = 0.020) and Δtotal C (*r* = –0.476, *P* = 0.008) (Figure 2, H and I). In parallel, we determined the magnitude effect of C16+18 SM, Δ%C20+22, and Δ%C24+26 Cer species on total C, LDL-C, and ApoB by estimating the regression coefficient associated to each variable in a general linear mixed model. This shows that each variation of 1 μM of ΔC16+18 SM species would result in a variation of 0.0074 mM of LDL-C (*P* = 0.0065), 0.0088 mM of total C (*P* = 0.0042), and 0.0017 g/L of ApoB (*P* = 0.017). The magnitude effect of Δ%C20+22 species on cardiovascular lipid markers was not significant, while each variation of 1% of ΔC24+26 Cer species proportions would result in a variation of 0.039 mM of LDL-C (*P* = 0.040), 0.054 mM of total C (*P* = 0.010), and 0.010 g/L of ApoB (*P* = 0.041).

### Milk PL decrease SM content in intestine-derived chylomicrons and impact their SM and Cer molecular profiles.

**Figure 3:**
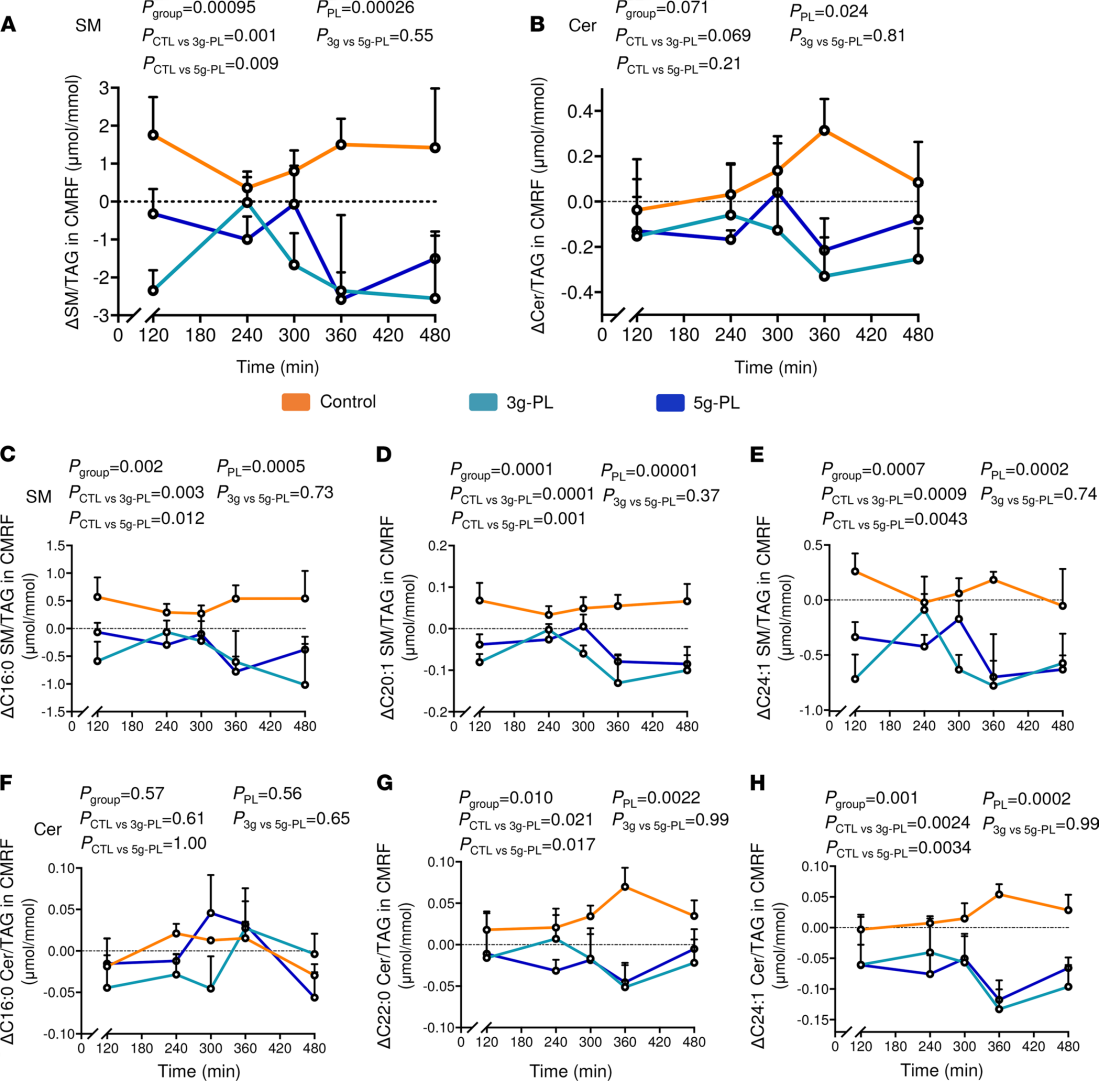
Milk PL supplementation during 4 weeks modulate SM and Cer molecular composition of plasma CMRF. (**A** and **B**) Kinetics of ΔV2–V1 CMRF SM and Cer normalized by CMRF TAG content. (**C**–**H**) Molecular composition analysis of specific SM and Cer species in CMRF after normalization by CMRF TAG content: C16:1 SM (**C**), C20:1 SM (**D**), C24:1 SM (**E**), C16:1 Cer (**F**), C22:0 Cer (**G**), and C24:1 Cer (**H**). Data are presented as mean ± SEM (*n* = 6 / group). The vertical dotted line represents the intake of the meal including the control or milk PL–rich dairy (according to group). The *P*_group_ and *P*_posthoc_ are shown for the postprandial period from 120 to 480 minutes. Statistical analysis was done using a linear mixed model followed by Tukey-Kramer’s post hoc test. *P*_posthoc_ corresponds altogether to *P*_CTL_ _versus_ _3g-PL_; *P*_CTL_ _versus_ _5g-PL_ and *P*_3g__versus_ _5g-PL_. Results are presented based on the assumption of sphingosine d18:1 as the major sphingoid base for determined SM and Cer species. Cer, ceramides; CMRF, chylomicron-rich fraction; CTL, control; PL, polar lipids; SM, sphingomyelins; TAG, triacylglycerols. See Supplemental Table 3 and Supplemental Figure 2, VALOBAB-C trial.

The variations of plasma concentrations of chylomicron-rich fraction–bound SM (CMRF-SM) decreased in the 5 g–PL group during all the postprandial period (*P*_group_ = 0.015; *P*_CTL-5g_ = 0.013), and the variation of plasma CMRF-Cer concentration also tended to decrease (*P*_group_ = 0.053; *P*_PL_ = 0.051) (Supplemental Table 3). To focus on potential modifications of chylomicron lipid composition regardless of their circulating concentration, we also determined their enrichment in SP by analyzing the SM/TAG and Cer/TAG ratios in CMRF particles. Milk PL significantly reduced the CMRF-SM/TAG ratio (Figure 3A; *P*_group_ = 0.00095; *P*_PL_ = 0.00026; *P*_CTL-3g_ = 0.001; *P*_CTL-5g_ = 0.009), notably after lunch that contained the test cream cheese (240–480 min). CMRF-Cer/TAG ratio also significantly decreased in milk PL–treated groups, regardless of dose (*P*_group_ = 0.071; *P*_PL_ = 0.024) (Figure 3B). Milk PL effects on SM molecular composition in intestine-derived chylomicrons were mainly mediated by a significant decrease in several CMRF-SM species content relative to CMRF-TAG including C16:0, C16:1, C18:0, C18:1, C20:1, C24:0, and C24:1 SM species (Figure 3, C–E, and Supplemental Figure 2). Changes in CMRF-Cer molecular composition was mainly driven by a decrease of C22:0 and C24:1 Cer species content relative to CMRF-TAG (Figure 3, F–H, and Supplemental Figure 2).

### Ileostomy model reveals an important increase of saturated SM and Cer species in ileal efflux.

**Figure 4:**
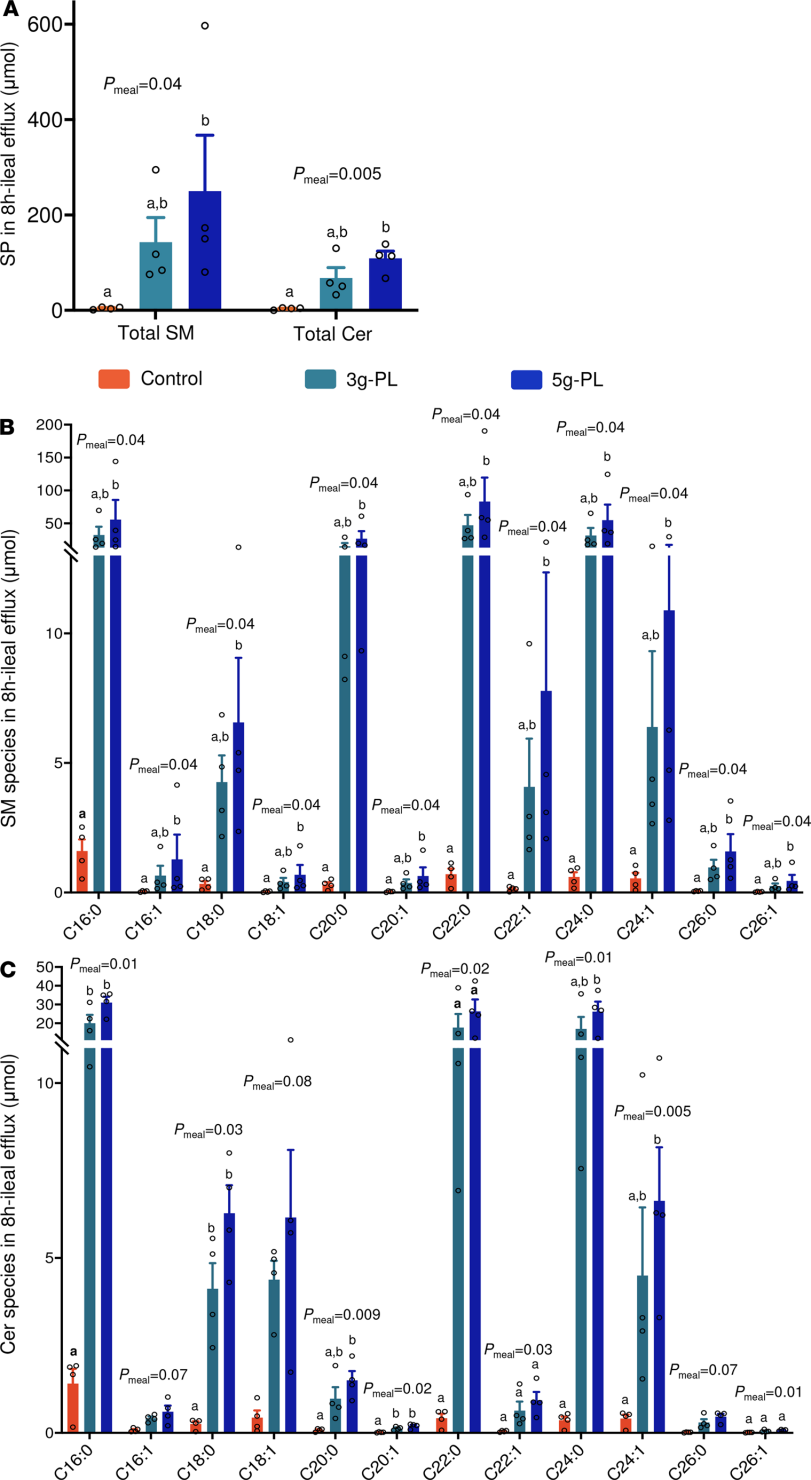
Milk PL ingestion modulates SM and Cer species in ileal efflux in ileostomy patients. (**A**) Cumulated enrichment over 0–480 minutes of total SM and Cer. (**B** and **C**) Molecular composition of ileal content efflux after 8 h of accumulation in SM (**B**) and Cer (**C**) species. Data are expressed in μmol and presented as mean ± SEM (*n* = 4/group), and empty circles represent individual values. Statistical analysis was done using 1-way ANOVA followed by Tukey’s post hoc test (normal data) or Friedman’s test followed by Dunn’s post hoc test (nonnormal data). Letters “a” and “b” indicate statistically different (*P* < 0.05) intervention effects between groups as calculated by post hoc analysis. Results are presented based on the assumption of sphingosine d18:1 as the major sphingoid base for determined SM and Cer species. Cer, ceramides; CTL, control; PL, polar lipids; SP, sphingolipids; SM, sphingomyelins. See Supplemental Figure 3, VALOBAB-D trial.

We performed a complementary mechanistic study in ileostomy patients to determine whether the digestive fate of milk PL in the upper gastrointestinal tract may contribute to the above results, notably before absorption and enterocyte metabolism (29). Each milk PL–enriched meal resulted in higher 8-hour cumulative ileal efflux of total SM (CTL: 4.4 ± 1.3 μmol; 3 g–PL: 143.2 ± 51.4 μmol; 5 g–PL: 250.2 ± 117.3 μmol; *P*_meal_ = 0.04) and Cer (CTL: 3.5 ± 1.0 μmol; 3 g–PL: 67.9 ± 21.5 μmol; 5 g–PL: 109.1 ± 15.0 μmol; *P*_meal_ = 0.005; Figure 4A). Detailed molecular composition analysis showed a significant increase in C16:0 SM (CTL: 1.6 ± 0.4 μmol; 3 g–PL: 32.2 ± 12.7 μmol; 5 g–PL: 55.7 ± 29.9 μmol; *P*_meal_ = 0.04), C20:0 SM (CTL: 0.3 ± 0.1 μmol; 3 g–PL: 15.2 ± 4.8 μmol; 5 g–PL: 26.7 ± 11.6 μmol; *P*_meal_ = 0.04), C22:0 SM (CTL: 0.7 ± 0.2 μmol; 3 g–PL: 46.9 ± 15.8 μmol; 5 g–PL: 83.3 ± 36.3 μmol; *P*_meal_ = 0.04), C16:0 Cer (CTL: 1.4 ± 0.4 μmol; 3 g–PL: 20.0 ± 4.4 μmol; 5 g–PL: 31.1 ± 3.1 μmol; *P*_meal_ = 0.01), C20:0 Cer (CTL: 0.1 ± 0.0 μmol; 3 g–PL: 1.0 ± 0.3 μmol; 5 g–PL: 1.5 ± 0.3 μmol; *P*_meal_ = 0.009), and C22:0 Cer species (CTL: 0.4 ± 0.1 μmol; 3 g–PL: 17.7 ± 7.2 μmol; 5 g–PL: 26.4 ± 6.3 μmol; *P*_meal_ = 0.02) (Figure 4, B and C). However, the analysis of SP species relative abundance revealed a reduction in the proportions of atherogenic C16:0 SM, C18:0 SM, and C24:1 Cer species (*P*_meal_ = 0.02, *P*_meal_ = 0.04, and *P*_meal_ = 0.04, respectively), and an increase in the proportions of C22:0 and C24:0 SM species (*P*_meal_ = 0.005 and *P*_meal_ < 0.001, respectively) and C22:0 and C24:0 Cer species (*P*_meal_ = 0.02 and *P*_meal_ = 0.04, respectively; Supplemental Figure 3, A and B). Considering that such lipids cannot be absorbed directly as such by enterocytes and that SM digestion is incomplete in the gastrointestinal tract (30), we explored the molecular composition of SM and Cer species in fecal samples collected by the postmenopausal women included in the VALOBAB-C trial.

### Fecal sphingolipidome is largely enriched in Cer after milk PL supplementation.

**Figure 5:**
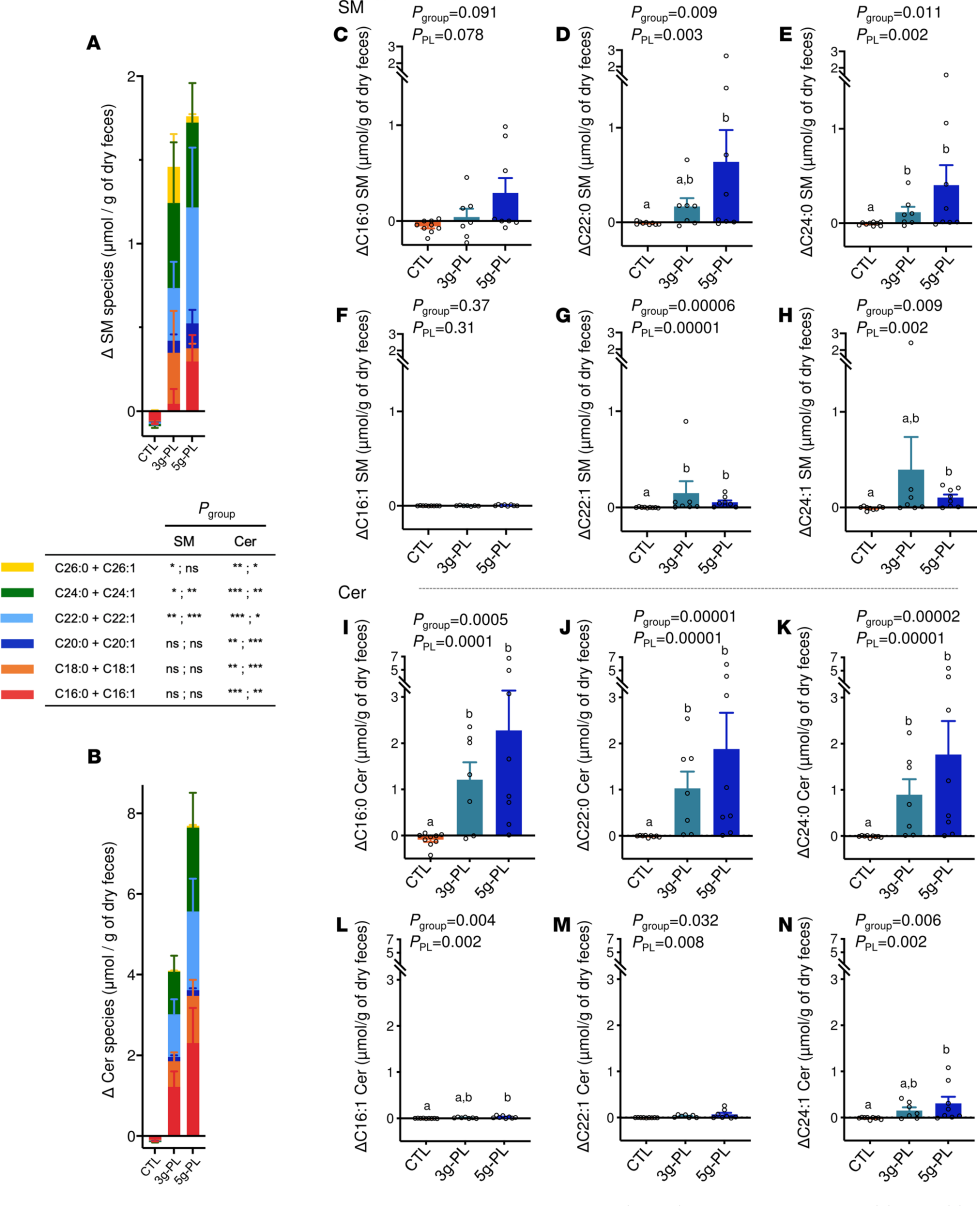
Effect of milk PL supplementation during 4 weeks on SM and Cer species excreted in feces. (**A** and **B**) Molecular composition of SM (**A**) and Cer (**B**) in fecal samples (ΔV2–V1). Data are presented as mean ± SEM (control, *n* = 9; 3 g–PL, *n* = 7; 5 g–PL, *n* = 8) and expressed in μmol/g of lyophilized feces. Empty circles represent individual values. (**C**–**M**) Variations of specific SP species present in fecal samples were also determined and expressed as percentage of total SM and Cer: C16:0 SM (**C**), C22:0 SM (**D**), C24:0 SM (**E**), C16:1 SM (**F**), C22:1 SM (**G**), C24:1 SM (**H**), C16:0 Cer (**I**), C22:0 Cer (**J**), C24:0 Cer (**K**), C16:1 Cer (**L**), C22:1 Cer (**M**), and C24:1 Cer species (**N**) (ΔV2–V1). Statistical analysis was done using nonparametric analysis (nonnormal data). Letters “a” and “b” indicate statistically different (*P* < 0.05) intervention effects between groups as calculated by post hoc analysis. Results are presented based on the assumption of sphingosine d18:1 as the major sphingoid base for determined SM and Cer species. Cer, ceramides; CTL, control; PL, polar lipids; SM, sphingomyelins. See Supplemental Table 4, VALOBAB-C trial.

The 4-week nutritional intervention significantly increased total fecal SM and Cer in milk PL supplemented groups compared with control (Figure 5, A and B) (ΔSM: CTL –0.09 ± 0.04 μmol; 3 g–PL +1.46 ± 0.95 μmol; 5 g–PL +1.76 ± 0.83 μmol/g of dry feces; *P*_group_ = 0.006; *P*_PL_ = 0.001; ΔCer: CTL -0.15 ± 0.08 μmol; 3 g–PL +4.09 ± 1.38 μmol; 5 g–PL +7.69 ± 2.95 μmol/g of dry feces; *P*_group_ = 0.0002; *P*_PL_ = 0.00006). Altogether, this increase of total fecal Cer was higher than that of SM (*P* = 0.015, ΔCer versus ΔSM in milk PL groups). The detailed molecular analysis revealed a major impact of intervention on saturated SP species — notably, an increase of C22:0 SM (*P*_group_ = 0.009; *P*_PL_ = 0.003), C24:0 SM (*P*_group_ = 0.011; *P*_PL_ = 0.002), C16:0 Cer (*P*_group_ = 0.0005; *P*_PL_ = 0.0001), C22:0 Cer (*P*_group_ = 0.00001; *P*_PL_ = 0.00001), and C24:0 Cer species (*P*_group_ = 0.00002; *P*_PL_ = 0.00001) (Figure 5, C–E, and I–K, and Supplemental Table 4). To a lower extent, the milk PL supplementation also increased the fecal amount of some unsaturated SP species (Figure 5, F–H and L–N, and Supplemental Table 4).

## Discussion

This study is the first to our knowledge to report how the daily consumption of a significant amount of dietary SP present in milk PL impacts the endogenous sphingolipidome in the bloodstream and along the gastrointestinal tract in humans. Firstly, we reveal that the increased intake of milk SM and Cer did not increase their total amount in serum, but the molecular composition of SM and Cer species was markedly improved by the 4-week intervention with milk PL. Notably, the atherogenic C16+18 SM and C24:1 Cer species decreased significantly, despite their increased intake from the provided supplementation. These variations even correlated with the beneficial impacts of milk PL on lipid cardiovascular markers reported previously (29). In addition, the Mayo Clinic published the reference values for circulating level of C24:1 Cer species (i.e., 0.65–1.65 μM; https://www.mayocliniclabs.com). Here, we found that serum C24:1 Cer species concentration returned within the normal range after the intervention in the 5 g–PL group only (V1, 1.96 ± 0.17 μM; V2, 1.59 ± 0.16 μM; *P*_group_ = 0.033). Our results demonstrate that milk PL supplementation positively impacts the endogenous sphingolipidome, with the specific decrease of serum SM and Cer species known for being associated with inflammation and metabolic disorders (31, 32). Previous studies reported that high concentrations of serum C18:0, C20:0, and C24:1 Cer species are associated with type 2 diabetes, while high serum levels of C16:0 Cer and C18:0 SM species correlate with insulin resistance (33). Regardless of milk PL dose, the analysis of the relative abundance of each SM and Cer species in the bloodstream revealed a significant increase in the proportions of C20:0 SM, C22:1 SM, and C20:0 Cer species that are normally poorly detected in human blood but found in nonnegligible amount in MFGM. We also estimated to what extent the changes in major SM or Cer species could explain the relationships between milk PL consumption and enhanced lipid cardiometabolic risk factors. According to the estimated regression coefficients, assuming a mean variation of C16+18 SM species of about –22 μM, as observed in the 5 g–PL group, its mean effect is expected to be (a) –0.16 mM on LDL-C (with a global effect of –0.34 mM observed in this group) (29) and (b) –0.19 mM on total C (with an observed effect of –0.4 mM). Moreover, assuming a mean variation of the relative proportions of C24+26 Cer of about –0.83% as observed in the 5 g–PL group, its mean effect is expected to be (a) –0.032 mM on LDL-C and (b) –0.045 mM on total C. These results show that changes in serum total C and LDL-C are significantly associated with changes in serum C16+18 SM species, and to a lower extent with the modulation of the relative proportion of C24+26 Cer species (here mainly driven by the variation of C24:1 Cer species).

To investigate underlying mechanisms involved in the effects of milk PL consumption on circulating SP species, we first estimated the contribution of intestine-derived chylomicrons, which are the dietary lipid carriers secreted by the small intestine during the postprandial phase. Chylomicrons represent a major source of circulating SM, although the mechanisms by which SM is inserted into these lipoproteins have not been established (34). Dietary SM and Cer are not absorbed as such; their lipolysis products released in the small intestine can be absorbed and a small proportion of their sphingoid bases contribute to the newly formed SP ultimately found in chylomicrons (34). Herein, the 4-week milk PL supplementation decreased chylomicron total SM and Cer, especially during the second part of the postprandial period (after test cheese consumption), without change in particle size (i.e., no change in the surface/TAG core ratio) (29). These modifications were also observed at species level for almost all SM and Cer species, including those whose concentrations in total serum decreased — namely C16:1 SM, C20:1 SM, and C24:1 Cer. Because SP are located at the surface of lipoproteins, this reveals a lower SP amount in the chylomicron composition. Whether this is due to decreased SM synthesis in enterocytes after intervention with dietary SP remains to be elucidated. Milk PL also modified the SM molecular profile in chylomicrons with an increase in the proportions of C20 SM species and a decrease in the proportions of C24:1 SM species — a possible precursor of C24:1 Cer via acid SMase (35). These results suggest that these modifications may originate from the gut or from enterocyte metabolism during the intestinal digestion and absorption processes.

To determine the contribution of SM and Cer in the intestine, we analyzed their molecular profiles in the gut lumen of ileostomy patients after the acute intake of milk PL, as well as in feces of the postmenopausal women after 4-week milk PL supplementation. These analyses revealed an increase of both total SM and Cer in gut contents in milk PL supplemented groups. At a molecular level, the amounts of most detected SP species of interest were significantly increased by milk PL consumption, especially C16:0, C22:0, C24:0 SM and Cer, and also C20:0 SM species. Altogether, SM and Cer species whose amounts increased in gut contents reflect species that are present in milk PL–enriched cheeses. These results are consistent with the fact that SM digestion is incomplete, as only 75%–80% of milk SM was reported to be digested and absorbed in humans (36). It has been previously reported that ileal efflux of C16:0 SM was only ~10% of ingested dose versus ~20% for C24:0 SM after intake of lower doses of SM — i.e., 50–200 mg (36) — suggesting that longer-chain saturated species of SM and Cer are less efficiently digested and absorbed. In addition, with the digestion of SM being slow and incomplete, it may induce an important increase of nondigested SM and nonabsorbed Cer in the lumen content (3, 30), which may explain the present results. Moreover, fecal metabolites, including the various lipid species normally found in feces, may originate directly from food, but also from host cells or bacterial cell components, or indirectly from the molecular conversion of SP by gut microorganisms or host enzymes (37). In a recent study performed in healthy patients, plasma and fecal lipidomic analyses demonstrated that the lipid fraction of fecal samples contains significant amounts of Cer species, with only 2 SM species detected, while plasma samples commonly contain significant amounts of several SM species and lower quantities of Cer (37, 38). Herein, we chose to determine the concentration of 12 SM and Cer species of interest in serum, and we were also able to quantify all these species in feces. In ileostomy patients, we report a higher total SM amount in ileal content compared with total Cer, while total Cer was largely more abundant in the fecal samples compared with total SM. The latter could be the result of several metabolic pathways such as the conversion of dietary SM species in Cer species by host enzymes present in the lumen and in enterocytes. At a molecular level, monounsaturated SM and Cer species increased in both ileal efflux and fecal samples, despite being found in minority in test cheeses compared with saturated species. A potential differential absorption process between monounsaturated and saturated species would, thus, deserve to be investigated. Interestingly, the major changes reported in the serum after the dietary intervention with milk PL mainly concern monounsaturated SM and Cer species. It may suggest that some modifications of serum and chylomicron SP profiles occur in response to changes in the SP fate in the small intestine.

The increased amount of total Cer reported in the fecal samples of milk PL–supplemented volunteers could also be the result of gut bacteria metabolism because several bacteria, including those belonging to the *Bacteroides* genus, were reported to be able to produce SP (39, 40). Very recently, Lee et al. demonstrated in female mice that sphinganine, which is the main sphingoid base of SM and Cer present in MFGM, is assimilated by gut bacteria (41). In this study, 99% of gavaged fluorescent sphinganine was assimilated by *Bacteroides* spp., with the remaining 1% by *Prevotella* spp., *Lactobacillus* spp., and *Bifidobacterium* genus (41). Also, *Bifidobacterium* spp., which are known to be increased after milk SM consumption in rodents (20, 26), can release free milk Cer by hydrolyzing milk gangliosides (42). In this context, the contribution of the gut microbiota SP metabolism in the effects of milk PL consumption on the intestinal and circulating SM and Cer profiles cannot be ruled out.

To further explore potential endogenous mechanisms, we analyzed whole blood cells gene expression of some key enzymes involved in SM synthesis (SM synthase 1 and 2 [SGMS1 and SGMS2, respectively]) and hydrolysis (an acid sphingomyelinase; SMase, also called SM phosphodiesterase 1 [SMPD1]). We found only slight effects on *SGMS1* and *SGMS2* expression (Supplemental Figure 4), while *SMPD1* expression decreased in milk PL–treated groups compared with control (*P*_group_ = 0.052; *P*_PL_ = 0.030). As previously described, acid SMase activity in plasma is increased in acute coronary syndromes (43). However, these results were not likely to explain the changes observed in the circulating sphingolipidome. It would also be relevant to consider the possible contribution of intestinal enzymes, given that the small intestine is rich in enzymes known to contribute to SP metabolism, such as alkaline SMase that converts SM in Cer (35). Unfortunately, we could not collect intestinal biopsies from healthy postmenopausal women in the present study. However, in an 8-week milk PL supplementation performed (0.9 g per 100 g of diet) in high-fat diet–fed mice (22), we observed a significant increase of jejunal expression of *Enpp7*, coding for the alkaline SMase, compared with the high-fat control group (1.7-fold change). Interestingly, previous preclinical studies reported opposite impacts of Cer production depending on SMase activity; Cer generated from neutral or acid intestinal SMases are more prompt to exert proinflammatory effects, while Cer generated from alkaline SMase promote antiinflammatory pathways (44, 45). The conversion of exogenous SM in Cer by the alkaline SMase could also play a role in the inhibition of cholesterol absorption (45, 46), which is concordant with the present findings and supports the role of SM metabolism in cholesterol absorption. Previous preclinical studies demonstrated that dietary SM are able to play a beneficial role on cholesterol levels and more largely in the prevention of cardiometabolic disorders (25, 26, 47, 48). In mice fed high-fat diets, supplementation with egg SM lowered intestinal absorption of cholesterol and lipids with a reduction of hepatic cholesterol (49). In vitro, both SM and Cer inhibit cholesterol absorption in Caco-2 intestinal epithelial cells (45). However, it has been suggested that small SM catabolites, such as Cer and sphingosine, might be the effectors of the beneficial impact of milk SM (2). The present findings consolidate our previous clinical results, given that the observed reductions in circulating total cholesterol, LDL-C, and ApoB (29) significantly correlate with the reduction of serum proinflammatory C16+18 SM in the milk PL–treated groups. In accordance with above-mentioned studies, our findings also bring potentially new information and insights on Cer in the gastrointestinal tract as signaling molecules potentially participating in the beneficial effect of milk PL consumption on cholesterol metabolism.

The present study has several strengths, but it also has some limitations that need to be outlined. The clinical trials were performed in a targeted population (i.e., overweight postmenopausal women) known to present an important risk of CVDs, but results cannot be extrapolated to other populations. We took care to include 4-day dietary records before and after the nutritional intervention to show that volunteers of the 3 groups did not differentially modify their energy and dietary intakes (Supplemental Table 5). Many parameters of the present study were measured in a limited number of subjects. Nevertheless, we performed, for the first time to our knowledge, a broad sphingolipidomic analysis, including a large scale of measurements of SM and Cer species at both fasting and postprandial states in various biological compartments — serum, chylomicron fractions, and feces — but also in 8 h-cumulative ileal efflux from ileostomy patients. The sphingolipidome is a complex and dynamic system that encompasses several important SP families, including dihydroceramides, gangliosides, and cerebrosides (17). Considering the variations in the sphingoid bases, FA, and headgroups of SP molecules, the number of species exceeds thousands. SP are localized in cellular membranes (lipid rafts) and are carried by albumin, lipoprotein particles, blood cells, and platelets in the bloodstream (50, 51). Based on the present findings, future studies should, thus, explore the sphingolipidome in other blood compartments and, potentially, epithelial cells to better understand the fate of milk SM and Cer species. Furthermore, we cannot exclude the potential contribution of other components of the PL fraction/MFGM from buttermilk concentrate and/or the lower milk TAG content in the PL-enriched cheeses in the reported metabolic effects in both trials. Putting aside these limitations, this is — to the best of our knowledge — the first time that such a wide sphingolipidomic analysis has been performed in humans in response to a controlled dietary intervention in the context of cardiometabolic disorders. The present study clearly responds to the need to identify relevant dietary strategies to improve the endogenous SP metabolism, which was highlighted in recent reviews (2, 16).

The present findings uncover that milk PL supplementation providing particular SP species markedly improved the endogenous sphingolipidome by reducing serum atherogenic C16+18 SM and C24:1 Cer species in overweight postmenopausal women at risk of CVD. These reductions in SP were (a) correlated with and (b) significantly involved in the decrease of lipid cardiovascular risk markers induced by milk PL intervention. We further demonstrate that, despite a significant ingestion of SP provided by milk PL, SM and Cer concentrations decreased in intestine-derived chylomicrons, while their concentration increased in gut contents. The related differences in SM and Cer profiles between gut contents and circulating compartments suggest that small intestinal mechanisms occurred during digestion and absorption processes of milk SM and Cer, and that a contribution of the gut microbiota may be possible. Considering that milk PL are naturally found in large amounts in buttermilk, which is still poorly valued in human food, such bioactive lipids could be envisioned as promising ingredients for the development of new functional foods, providing health effects in the frame of chronic diseases.

## Methods

### VALOBAB-C trial.

Details of the VALOBAB-C study have been published previously (29). Briefly, the multicenter study used a double-blind randomized placebo-controlled parallel design and was conducted in 58 overweight postmenopausal women, without metabolic syndrome but at risk of CVD. The eligibility criteria and sample size calculation have been described previously (29). Volunteers were randomly divided into 3 groups. Randomization was performed electronically using a random number generator and supervised by a biostatistician (29). Both volunteers and investigators were kept blind regarding group allocation. Volunteers were subjected to the daily consumption of either control or milk PL–enriched cream cheese (100 g of cream cheese containing 13 g of total fat including 0 [control], 3, or 5 g–milk PL during 28 days; *n* = 19, 19, and 20, respectively). The strategic approach was to formulate cheeses with identical total lipid content with partial substitution of TAG by milk PL to avoid increased energy intake. The 3 g– and 5 g–PL cream cheeses were based on a butterserum concentrate rich in milk PL prepared according to Gassi et al. (52) representing a 3- to 5-fold increased daily consumption of milk SM and Cer compared with an estimated intake of dairy SP in Western countries (Supplemental Table 1) (17). After the “run-in” period, volunteers were subjected to a first exploratory visit (V1), followed by 28 days of intervention and ended by a second exploratory visit (V2). During each visit, overnight-fasted participants received a breakfast meal rich in fat and carbohydrates, and 4 hours later, they consumed a standardized lunch containing the corresponding test cream cheese, thus dividing the exploratory visit in 2 specific postprandial periods (0–240 min and 240–480 min, as detailed previously) (29). Volunteers were asked to continue their usual diet and physical activity throughout the study. Participants were told to avoid the consumption of cheeses other than the test cream cheese and to avoid listed foods that may influence the gut microbiota composition. Particular attention was drawn to standardize the meal consumed the evening before each postprandial exploratory visit. Subjects recorded their food consumption for 4 days before and after the nutritional intervention. No difference in changes in energy and macronutrient intakes, fibers, alcohol, or cholesterol or FA intakes was observed between groups (29). The primary outcome was the impact of the 4-week milk PL consumption on fasting serum concentration of total C (29). The predefined secondary outcomes tested in the present study were related to the impact of the dietary intervention on serum, chylomicron, and fecal SM and Cer profiles. Considering available samples and practical/technical aspects, some analyses were performed on a subgroup of individuals only (Figure 1).

### VALOBAB-D trial.

The double-blind randomized controlled trial (RCT) was performed in 4 ileostomy patients following a crossover design, as previously described (29) (Supplemental Figure 5). An ileostomy is a surgical opening in the abdomen in which a piece of the ileum is brought outside the abdominal wall to create a stoma through which digestive contents leave the body and are collected in a pouch (ileal efflux). Selected patients according to eligibility criteria were invited to participate in 3 distinct exploratory visits separated by a 4- to 6-week washout period (29). During each visit, overnight-fasted patients consumed one of the test cream cheese containing 0, 3, or 5 g–milk PL, and their ileal efflux was collected over 8 hours. Sequences of meal allocation were based on a random number generator (29). Both patients and investigators were kept blind regarding meal allocation.

### Isolation of CMRF.

Isolation of intestine-derived CMRF was performed by ultracentrifugation from plasma collected at different time points, as previously described (29, 53).

### Analysis of serum phospholipids.

Total lipids were extracted from 300 µL of serum with chloroform/methanol (2:1, v/v) according to the method of Folch et al. (54). After drying under nitrogen, total lipids were determined gravimetrically and were dissolved precisely with 1 mL of chloroform/methanol (2:1, v/v). This stock solution of total lipids was stored at –20°C. Phospholipid classes were then separated by high-performance liquid-chromatography (HPLC) coupled to an evaporative light-scattering detector (SEDEX LT-ELSD SOLT, HPLC DDL Sedere, Thermo Fisher Scientific) (55, 56), using a silica normal-phase column (Lichrospher Si 60, 3 μm, 100 × 4.6 mm, Waters). The chromatographic separation was carried out using a linear binary gradient according to the following scheme: t0 minutes: 90%A, 10%B 0%C; t20 minutes: 42%A, 52%B, 6%C; t30 minutes: 32%A, 52%B, 16%C; t55 minutes: 30%A, 70%B, 0%C; t60 minutes: 90%A, 10%B, 0%C. Total chromatographic run time was 75 minutes per sample, which consisted of a 60-minute analysis and 15 minutes to restore initial conditions and reequilibration. Eluent A consisted of hexane/tetrahydrofuran (99:1, v/v), eluent B of isopropanol/chloroform (80:20, v/v/v), and eluent C of isopropanol/water (50:50, v/v/v). The flow rate of the eluent was 1 mL/min. Identification of phospholipids and lysophospholipids was carried out by comparison with the retention time of pure standards (Avanti polar Lipids). Calibration curves for each compound were calculated from the area values of stock solution of pure standards between 0.1 and 1 mg/mL. Results were analyzed using Chromeleon software (Thermo Fisher Scientific) and expressed as μg/100 μL of serum.

### Analysis of SM and Cer molecular profiles.

Concentrations of SM and Cer molecular species of interest were determined in serum, CMRF, ileal efflux, and fecal samples and also in test cream cheese, according to the method by Kyrklund (57), which was optimized as previously described (27). Ileal content from patients with ileostomy and fecal samples obtained from VALOBAB-C trial’s volunteers were freeze-dried, and approximately 15–40 mg of lyophilized matter, accurately weighted, were dissolved in 1 mL of apyrogen water prior to lipid extraction. Briefly, for each sample, total lipids were extracted using 2.5 mL of chloroform/methanol (1:2 v/v) in the presence of 2 deuterium-labeled internal standards (N-heptadecanoyl-D-erythro-sphingosine [C17:0-Ceramide]; N-palmitoyl[d31]-D-erythro-sphingosylphosphorylcholine [C16:0D31SM] from Avanti Polar Lipids). After 2 hours of shaking and centrifugation (room temperature, 10 minutes, 1900*g*), samples were evaporated with liquid nitrogen. The dry samples were dissolved in 1.5 mL of chloroform/methanol (1:2 v/v) and sonicated 30 seconds on ice. SP were then isolated by saponification with potassium hydroxide for 2 hours at 37°C and then fractionated and desalted using reverse-phase Bond Elut C18 columns. The final elutions were done with 2 × 1 mL of chloroform/methanol (12:1 v/v) and 2 × 1 mL of chloroform/methanol (1:2 v/v) prior to the evaporation of samples with liquid nitrogen. The dry extracts were kept at –20°C until tandem mass spectrometry analysis (MS/MS). Samples were homogenized in 1 mL of chloroform/methanol (1:2 v/v) and analyzed by direct flow injection on a triple-quadrupole mass spectrometer (API 4500 QTRAP MS/MS; Sciex Applied Biosystems) in the positive ionization mode using the multiple reaction monitoring (MRM) method. Cer and SM species were measured separately, with 2 different methods with a flow rate of 200 μL/min (analysis time of 3 minutes). We quantified 12 species of SM and of Cer that are (a) the most abundant in human, (b) of particular interest in cardiovascular risk, and (c) also found in bovine milk in different proportions (Table 1). The concentration of each molecular species was calculated from the ratio of its signal to that of the corresponding internal standard. Total Cer and SM concentrations were the sum of the concentrations of the various species. Results are presented based on the assumption of sphingosine d18:1 as the major sphingoid base for determined SM and Cer species. These analyses were performed on a MS/MS platform accredited following EN NF ISO 15189 requirements. The coefficient of variation (CV) for total SM and Cer was 4.4% and 5.4%, respectively. For the most abundant isoforms (C16:0, C22:0, C24:0, and C24:1 SM/Cer), the average CV was 7% ± 4%. The CV for the less abundant isoforms is slightly higher: 17% ± 5% for Cer and 9% ± 6% for SM species. These elements are in agreement with the Methods and Protocols section of LIPIDMAPS for the analysis of SP (58).

### Gene expression analysis in whole blood cells.

The PAXgene Fresh Whole Blood RNA samples were processed using the PAXgene Blood RNA Kit based on column purification of nucleic acids (PreAnalytiX, QIAGEN) as previously described (29). After reverse transcription, real-time PCR assays of *SGMS1* (forward [F], 5′ - CCTGGTATGCATTTCAACTG - 3′; reverse [R], 5′ - TGGCCGCTGTACAGATAGTC - 3′), *SGMS2* (F, 5′ - CAATAGTGGGACGCAGATTC - 3′ and R, 5′ - GGACAATCCACCACCAGAAA - 3′), and *SMPD1* (F, 5′ - CATCCTGCCAGGTTACATCG - 3′; R, 5′ - CACACCTCCACCATGTCATC - 3′) were assessed using a Rotor-Gene 6000 (QIAGEN), and obtained values were normalized to the expression of the housekeeping gene *PGK1* (phosphoglycerate kinase 1; F, 5′ - CCATGGTAGGAGTCAATCTG - 3′; R, 5′ - AGCTGGATCTTGTCTGCAAC - 3′).

### Data sharing.

Data of this study are protected under the protection of health data regulation set by the French National Commission on Informatics and Liberty (Commission Nationale de l’Informatique et des Libertés, CNIL). According to French law on the publication of biomedical research/clinical trials, we are not allowed to make the clinical database publicly available on the web, nor to make visible the location of the study associated with the database, nor to send the clinical database to third parties. The anonymized data that support the findings of the present study can be available upon reasonable request to the corresponding author. The French law forbids us to provide open access to raw data; access could, however, be given after legal verification of the use of the anonymized data.

### Statistics.

Regarding VALOBAB-C, continuous variables are described as mean ± SEM. The 4-week intervention impact was determined by comparing the variation of each variable between exploratory visits (i.e., ΔV2–V1) between groups (*P*_group_) (i.e., control versus 3 g–PL versus 5 g–PL group; Figure 1). Single time point parameters were analyzed through a general linear model and a subsequent Tukey’s post hoc test. *P*_posthoc_ corresponds altogether to *P*_CTL_ _versus_ _3g-PL_; *P*_CTL_ _versus_ _5g-PL_ and *P*_3g__versus_ _5g-PL_ as mentioned in the text and figures. For parameters analyzed along the postprandial period, a mixed linear modeling (MIXED procedure) was performed to account for within-subject repeated measures, seeking for main effects, at least “group” or time effect and interaction. Post hoc analyses were performed following Tukey-Kramer’s test to both detail main effects and control for familywise type I error. In case of residual distribution departing from normality, the analyses were performed on ranks. Global “milk PL” effect was also considered as binary factor, and statistical analysis was performed by lumping together milk PL doses in one group versus control. Spearman’s correlation analyses were also performed between blood lipid markers of cardiovascular risk and serum SM and Cer species grouped in 3 subclasses (i.e., C16+C18, C20+C22, and C24+C26 SM or Cer species). In order to check for any confounding effect, these analyses were also carried out adjusting for center, age, and waist circumference quartiles. Analyses were performed on SAS v9.4 (SAS Institute Inc. ) with a 2-sided type I error set at 0.05. In order to determine to what extent the changes in SM and Cer species could explain their relationship with lipid markers of cardiometabolic risk, we performed additional analyses. We aimed to adjust the analysis of ΔLDL-C, Δtotal C, and ΔApoB variables with ΔC16+18 SM, Δ%C20+22 Cer, and Δ%C24+26 Cer species variables. We first transformed each covariate as a 4 classes ordinal variable and then checked for a linear relationship between each covariate and each response variable, seeking an almost constant effect from an ordinal class to its neighbor. Since we merely found a monotonic relationship, it allowed us to include these covariates in their original continuous form, associated with a unique and relevant regression coefficient, thereby simplifying interpretation. We then reported the magnitude of the effect of ΔC16+18 SM, Δ%C20+22, and Δ%C24+26 Cer species on Δtotal C, ΔLDL-C, and ΔApoB by estimating the coefficient of regression associated to each variable in the mixed linear general model.

Regarding VALOBAB-D, data are presented as mean ± SEM and were analyzed with GraphPad Prism 8.3. For normally distributed data (Shapiro-Wilk’s test), repeated measures 1-way ANOVA was performed followed by Tukey’s post hoc test. For nonnormally distributed data, a Friedman’s test was performed followed by Dunn’s post hoc test. The variation between groups was reported using *P*_meal_ values, and post hoc analyses were added directly on corresponding figures using the letters a and b.

For all analyses performed in both clinical trials, results were considered significant for *P* < 0.05.

All graphs and heatmaps were created using GraphPad Prism 8.3.

### Study approval.

Both clinical trials were approved by the Scientific Ethics Committee of Lyon Sud-Est-IV and ANSM (French Agency for the Safety of Health Products) and registered at Clinical Trials (NCT02099032, NCT02146339). The clinical trials were conducted at the Human Nutrition Research Centre Rhône-Alpes (CRNH-RA; Lyon, France) and at the Human Nutrition Research Centre Auvergne (CRNH-A; Clermont-Ferrand, France) according to the Second Declaration of Helsinki and the French Huriet-Serusclat law. All data reported in the current article were obtained from samples stored in the biobank during the clinical studies, for which participants gave a written consent prior to inclusion in the study to use the samples for further metabolic analyses. All authors had access to the study data and reviewed and approved the final manuscript.

## Author contributions

MLB contributed conceptualization, validation, formal analysis, investigation, data curation, writing of the original draft, and visualization; CV contributed conceptualization, methodology, validation, formal analysis, investigation, data curation, writing of the original draft, and visualization. E. Combe and LJC contributed methodology, formal analysis, investigation, and data curation. M. Lecomte contributed conceptualization, validation, formal analysis, investigation, and data curation. FJ contributed methodology, validation, formal analysis, and investigation. MT, SP, EL, AEB, KB, JD, AD, and CC contributed investigation. EM contributed methodology, validation, and investigation. CR contributed formal analysis and data curation. PG, NL, and E. Cotte provided essential resources. ABD contributed validation, formal analysis, and investigation. M. Laville contributed conceptualization. SLP contributed conceptualization, methodology, validation, and investigation. LO contributed methodology, formal analysis, and data curation. HV contributed to results interpretation and revised the manuscript. CMB contributed conceptualization and methodology. DC contributed conceptualization, methodology, validation, formal analysis, investigation, writing of the original draft, and data visualization. MCM contributed conceptualization, methodology, writing of the original draft, data visualization, project administration, and supervision and takes primary responsibility for final article content. All authors read, revised, and approved the final manuscript.

## Supplementary Material

Supplemental data

Trial reporting checklists

ICMJE disclosure forms

## Figures and Tables

**Table 1:**
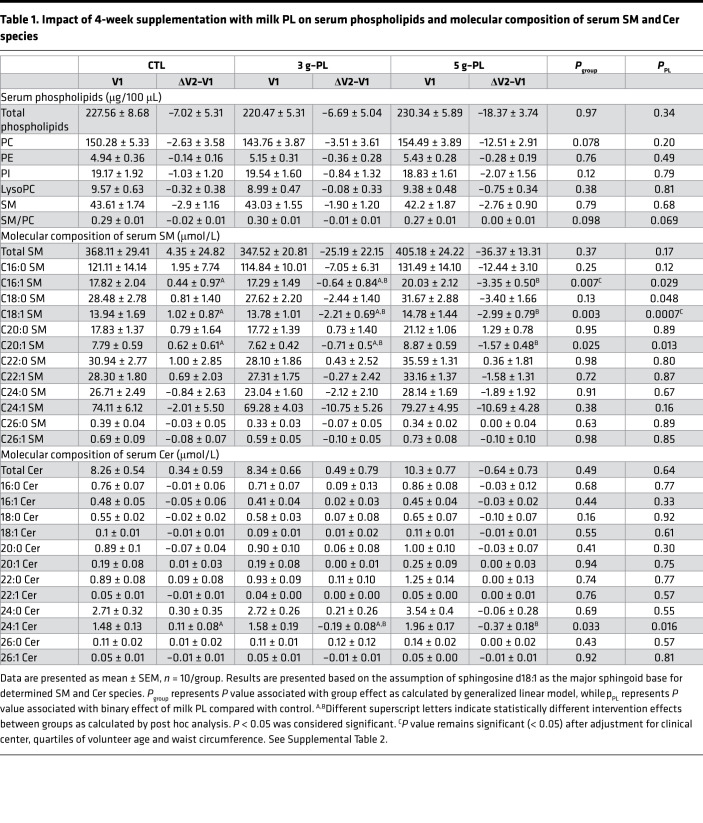
Impact of 4-week supplementation with milk PL on serum phospholipids and molecular composition of serum SM and Cer species

## Version 1 04/15/2021

In-Press Preview

## Version 2 05/24/2021

Electronic publication
