# Supplementary material for: Milk polar lipids favorably alter circulating and intestinal ceramide and sphingomyelin species in postmenopausal women
Source: JCI Insight. 2021 May 24;6(10):e146161. doi: 10.1172/jci.insight.146161 (PMC8262315; doi:10.1172/jci.insight.146161)
Supplement: Supplemental data [file jciinsight-6-146161-s276.pdf]

Supplemental Table 1. SM and Cer species of interest in control and milk PL-enriched cream cheeses.

Species		Control	3g-PL	5g-PL	Relative proportion (%)
		µmol / 100 g of cheese			
SM	C16:0	3.4	101.4	128.6	26.3
	C16:1	< 0.1	2.7	3.3	< 1
	C18:0	0.4	10.9	13.6	2.8
	C18:1	< 0.1	1.6	1.9	< 1
	C20:0	1.4	47.2	59.4	12.2
	C20:1	< 0.1	1.3	1.6	< 1
	C22:0	3.4	117.4	147.5	30.3
	C22:1	0.5	15.9	19.7	4.1
	C24:0	1.8	69.0	86.1	17.8
	C24:1	0.6	17.4	21.5	4.5
	C26:0	< 0.1	1.9	2.4	< 1
	C26:1	< 0.1	0.7	0.8	< 1
Cer	C16:0	0.2	3.5	6.4	29.7
	C16:1	< 0.1	< 0.1	< 0.1	< 1
	C18:0	< 0.1	0.3	0.6	2.7
	C18:1	< 0.1	< 0.1	< 0.1	< 1
	C20:0	< 0.1	0.2	0.3	1.5
	C20:1	< 0.1	< 0.1	< 0.1	< 1
	C22:0	0.2	3.2	6.0	27.5
	C22:1	< 0.1	0.1	0.2	< 1
	C24:0	0.3	3.6	6.4	30.1
	C24:1	< 0.1	0.7	1.2	5.8
	C26:0	< 0.1	< 0.1	0.1	< 1
	C26:1	< 0.1	< 0.1	< 0.1	< 1

Results are presented based on the assumption of sphingosine d18:1 as the major sphingoid base for determined SM and Cer species. Cer: ceramides; PL: polar lipids; SM: sphingomyelin.
Nota Bene: Cream cheese macronutrient and fatty acid composition is described in Vors et al. Gut 2020 (Supplemental Table 1) – all cheeses were isocaloric and contained similar amounts of total fat (13g/100g), proteins and carbohydrates.

Supplemental Table 2. Effect of milk SP *via* milk PL supplementation on serum SM and Cer molecular profiles (complementary to Table 1, VALOBAB-C trial).

%		Control		3g-PL		5g-PL		P_{group}	P_{PL}
		V1	ΔV2-V1	V1	ΔV2-V1	V1	ΔV2-V1		
SM	**C16:0**	32.17±2.03	0.58±0.42	32.79±1.57	0.35±0.66	31.87±1.89	-0.22±0.69	0.63	0.49
	**C16:1**	4.75±0.3	0.11±0.1[a]	4.96±0.31	0.23±0.14[a]	4.88±0.31	-0.42±0.14[b]	**0.003†**	0.29
	**C18:0**	7.73±0.43	0.05±0.22	7.91±0.28	-0.17±0.18	7.75±0.35	-0.26±0.19	0.53	0.27
	**C18:1**	3.75±0.31	0.2±0.13[a]	3.98±0.24	-0.4±0.13[b]	3.59±0.18	-0.49±0.14[b]	**0.002†**	**0.0001†**
	**C20:0**	4.9±0.19	0.14±0.14[a]	5.08±0.2	0.62±0.08[b]	5.3±0.25	0.95±0.09[b]	**0.00005†**	**0.0004†**
	**C20:1**	2.15±0.11	0.12±0.08[a]	2.2±0.08	-0.01±0.08[a.b]	2.2±0.12	-0.2±0.07[b]	**0.018**	**0.026**
	**C22:0**	8.45±0.37	0.18±0.29	8.14±0.46	0.67±0.21	8.95±0.41	0.99±0.33	0.14	0.067
	**C22:1**	7.9±0.43	-0.02±0.1	7.92±0.44	0.65±0.27	8.35±0.43	0.56±0.23	0.07	**0.021**
	**C24:0**	7.29±0.35	-0.28±0.29	6.65±0.33	-0.2±0.26	7.05±0.4	0.07±0.28	0.64	0.53
	**C24:1**	20.61±1.42	-1.04±0.23	20.08±0.83	-1.71±0.33	19.81±1.23	-0.97±0.47	0.30	0.51
	**C26:0**	0.11±0.01	-0.01±0.01	0.09±0.01	-0.02±0.01	0.09±0.01	0.01±0.01	0.32	0.75
	**C26:1**	0.19±0.03	-0.03±0.01	0.17±0.02	-0.02±0.01	0.18±0.02	-0.01±0.02	0.75	0.47
Cer	**C16:0**	9.22±0.63	-0.45±0.47	8.58±0.04	0.17±0.40	8.38±0.59	0.29±0.85	0.66	0.36
	**C16:1**	5.90±0.64	-0.85±0.65	5.09±0.42	0.24±0.35	4.41±0.42	0.04±0.28	0.23	0.09
	**C18:0**	6.78±0.39	-0.64±0.44	7.16±0.31	0.21±0.35	6.57±0.85	-0.67±0.79	0.46	0.56
	**C18:1**	1.26±0.16	-0.15±0.13	1.16±0.15	0.00±0.14	1.08±0.12	-0.01±0.09	0.64	0.34
	**C20:0**	11.12±1.27	-1.44±0.65	11.11±1.05	0.38±0.56	9.82±0.90	0.47±0.60	0.057	**0.016**
	**C20:1**	2.45±1.02	-0.05±0.32	2.16±0.81	0.07±0.18	2.37±0.81	0.13±0.24	0.88	0.63
	**C22:0**	10.71±0.65	0.62±0.75	11.14±0.52	0.39±0.25	12.00±0.65	0.61±0.53	0.95	0.86
	**C22:1**	0.60±0.06	-0.09±0.07	0.53±0.05	-0.05±0.04	0.46±0.05	-0.02±0.06	0.74	0.47
	**C24:0**	32.26±2.25	2.39±1.72	32.50±1.44	0.83±1.23	34.13±1.93	1.85±0.94	0.95	0.52
	**C24:1**	17.76±0.92	0.65±0.76[a]	18.54±0.95	-2.67±0.96[b]	18.88±0.47	-2.65±0.71[b]	**0.01**	**0.002†**
	**C26:0**	1.33±0.16	0.08±0.16	1.41±0.15	0.57±0.59	1.38±0.19	0.06±0.13	0.54	0.60
	**C26:1**	0.61±0.09	-0.10±0.08	0.63±0.05	-0.15±0.05	0.53±0.05	-0.09±0.06	0.81	0.83

Data are presented as mean ± SEM and expressed as the percentage of each SM or Cer species in total analyzed serum SM or Cer, respectively, n = 10 / group. Results are presented based on the assumption of sphingosine d18:1 as the major sphingoid base. *P* values presented in bold highlight significant intervention effect. P_{group} represents *P* value associated with group effect as calculated by generalized linear model, while P_{PL} represents *P* value associated with binary effect of milk PL compared to control. † *P* value remains significant (< 0.05) after adjustment for clinical center, quartiles of volunteer age and waist circumference. [a.b] Different superscript letters indicate statistically different intervention effects between groups as calculated by *post hoc* analysis (Tukey-Kramer's test). Cer: ceramides; PL: polar lipids; SM: sphingomyelin.

Supplemental Table 3. Kinetics of CMRF total SM and Cer before and after milk PL supplementation during 4 weeks (complementary to Figure 3 and Supplemental Figure 2, VALOBAB-C trial).

			SP species in CMRF			
		min	µmol / L plasma		µmol / mmol of CMRF TAG	
			V1	V2	V1	V2
Total SM	CTL	*120*	7.91 ± 1.07	10.65 ± 2.53	9.32 ± 0.81	11.08 ± 1.60
		240	13.29 ± 2.03	15.84 ± 2.52	9.35 ± 1.66	9.72 ± 1.54
		300	15.55 ± 2.36	18.53 ± 2.56	8.61 ± 0.83	9.42 ± 0.85
		360	14.36 ± 2.35	17.14 ± 3.40	11.78 ± 1.12	14.03 ± 1.40
		480	8.51 ± 1.40	9.66 ± 0.92	12.33 ± 1.29	13.74 ± 1.64
	3g-PL	*120*	14.33 ± 4.46	12.44 ± 4.39	15.14 ± 3.18	12.79 ± 2.87
		240	21.44 ± 5.81	23.08 ± 8.62	13.85 ± 3.48	13.92 ± 3.04
		300	27.41 ± 8.66	22.62 ± 7.35	12.28 ± 1.40	10.62 ± 2.20
		360	22.64 ± 9.27	25.77 ± 11.70	18.14 ± 3.54	15.76 ± 2.12
		480	18.28 ± 9.21	21.07 ± 11.52	17.15 ± 2.17	14.58 ± 1.62
	5g-PL	*120*	11.31 ± 2.12	10.09 ± 1.64	11.37 ± 0.71	11.05 ± 0.90
		240	16.95 ± 3.69	14.08 ± 2.99	12.25 ± 1.48	11.82 ± 1.44
		300	19.95 ± 4.45	13.95 ± 3.59	10.60 ± 1.15	10.53 ± 0.92
		360	18.85 ± 4.78	12.56 ± 3.86	14.65 ± 1.34	12.07 ± 1.34
		480	13.62 ± 3.78	9.43 ± 2.54	15.49 ± 1.63	13.98 ± 1.95
Total Cer	CTL	*120*	1.14 ± 0.10	1.23 ± 0.09	1.43 ± 0.21	1.40 ± 0.12
		240	1.82 ± 0.29	2.04 ± 0.24	1.19 ± 0.12	1.23 ± 0.07
		300	2.11 ± 0.41	2.45 ± 0.42	1.12 ± 0.09	1.26 ± 0.17
		360	1.98 ± 0.49	2.35 ± 0.42	1.61 ± 0.15	1.90 ± 0.13
		480	1.06 ± 0.21	1.21 ± 0.21	1.54 ± 0.25	1.62 ± 0.17
	3g-PL	*120*	2.58 ± 1.13	2.27 ± 0.83	2.22 ± 0.24	2.07 ± 0.33
		240	3.53 ± 1.22	3.46 ± 1.30	1.92 ± 0.28	1.87 ± 0.35
		300	4.28 ± 1.51	4.00 ± 1.38	1.83 ± 0.22	1.71 ± 0.34
		360	3.76 ± 1.69	3.73 ± 1.50	3.04 ± 0.69	2.71 ± 0.73
		480	2.30 ± 1.08	2.77 ± 1.26	2.37 ± 0.27	2.12 ± 0.18
	5g-PL	*120*	1.43 ± 0.17	1.28 ± 0.20	1.63 ± 0.22	1.50 ± 0.25
		240	2.14 ± 0.47	1.69 ± 0.46	1.56 ± 0.13	1.31 ± 0.12
		300	2.72 ± 0.61	1.85 ± 0.57	1.47 ± 0.17	1.51 ± 0.31
		360	2.37 ± 0.61	1.60 ± 0.41	1.90 ± 0.22	1.69 ± 0.17
		480	1.47 ± 0.32	1.12 ± 0.21	1.85 ± 0.16	1.77 ± 0.22

Data are presented as mean ± SEM (n = 6 / group) and correspond to total analyzed SM and Cer in intestine-derived chylomicrons before (V1) and after (V2) the 4-week nutritional intervention with milk PL. Raw results are expressed in µmol / L of plasma (Total SM: P_{group}=0.015, P_{PL}=0.025, $P_{CTL\ vs\ 3g\text{-}PL}$=0.53, $P_{CTL\ vs\ 5g\text{-}PL}$=0.013, $P_{3g\ vs\ 5g\text{-}PL}$=0.10; Total Cer: P_{group}=0.053, P_{PL}=0.051, $P_{CTL\ vs\ 3g\text{-}PL}$=0.58, $P_{CTL\ vs\ 5g\text{-}PL}$=0.045, $P_{3g\ vs\ 5g\text{-}PL}$=0.25) and were normalized by CMRF TAG plasma concentration. ΔV2-V1 kinetic curves are presented in the Figure 3A and B with corresponding statistical analysis. Cer: ceramides; CMRF: chylomicron-rich fraction; CTL: control; PL: polar lipids; SP: sphingolipids; SM: sphingomyelins; TAG: triacylglycerols.

Supplemental Table 4. Impact of milk SP *via* milk PL supplementation during 4 weeks on SM and Cer species in feces of postmenopausal women (complementary to Figure 5, VALOBAB-C trial).

nmol/g		CTL		3gPL		5gPL		P_{group}	P_{PL}
		V1	ΔV2-V1	V1	ΔV2-V1	V1	ΔV2-V1		
SM	C16:0	77.9 [42.5; 151.3]	-40.2 [-75.8; 0.7]	57.0 [27.6; 262.1]	-14.5 [-160.8; 155.5]	57.4 [29.2; 86.2]	11.1 [-7.7; 706.6]	0.091	0.078
	C16:1	2.3 [2.1; 4.3]	-0.2 [-1.8; 0.7]	1.7 [1.2; 8.1]	0.2 [-3.5; 2.4]	1.0 [0.6; 2.7]	1.1 [0.0; 12.0]	0.37	0.31
	C18:0	12.7 [7.4; 15.7]	-1.4 [-11.7; 5.3]	11.3 [4.3; 78.7]	4.5 [-52.3; 55.3]	16.4 [4.5; 27.5]	0.3 [-7.9; 105.0]	0.53	0.28
	C18:1	3.4 [2.3; 5.6]	0.2 [-3.0; 2.6]	2.2 [1.0; 7.4]	5.5 [-1.8; 216.5]	2.7 [1.3; 4.8]	15.0 [5.6; 65.6]	0.12	**0.043**
	C20:0	8.0 [4.7; 14.4]	-2.0 [-6.0; 1.1]	8.4 [5.5; 18.3]	4.6 [-6.3; 70.6]	8.4 [4.2; 11.4]	25.7 [-0.6; 219.2]	0.063	**0.026**†
	C20:1	2.0 [1.5; 4.0]	-0.2 [-0.9; 4.2]	2.1 [0.8; 3.7]	5.0 [-0.5; 32.5]	1.9 [1.3; 2.7]	2.9 [0.5; 12.1]	0.24	0.089
	C22:0	23.7 [18.5; 35.0]	-9.6 [-20.3; 0.8][a]	16.1 [11.3; 54.9]	173.1 [-6.1; 177.8][a,b]	14.4 [9.5; 22.4]	162.7 [6.8; 1066.7][b]	**0.009**†	**0.003**†
	C22:1	4.0 [2.6; 8.4]	-0.8 [-2.9; 0.2][a]	3.2 [1.6; 11.2]	14.6 [6.4; 57.1][b]	2.0 [1.5; 7.8]	37.4 [9.2; 82.1][b]	**0.00006**†	**0.00001**†
	C24:0	14.7 [9.3; 40.1]	-1.9 [-21.6; 4.7][a]	20.0 [10.2; 49.3]	88.9 [7.2; 194.2][b]	13.7 [10.8; 16.8]	84.6 [10.1; 736.5][b]	**0.011**†	**0.002**†
	C24:1	19.0 [7.0; 31.9]	-0.4 [-19.0; 0.2][a]	21.1 [7.6; 53.7]	30.9 [-2.7; 187.2][a,b]	8.0 [5.7; 15.5]	90.0 [9.0; 192.4][b]	**0.009**†	**0.002**†
	C26:0	2.0 [0.8; 3.2]	-1.5 [-2.0; 0.3][a]	2.2 [0.4; 2.6]	2.4 [-1.7; 6.9][a,b]	1.2 [0.8; 1.8]	4.3 [0.2; 20.0][b]	**0.024**†	**0.010**†
	C26:1	1.4 [0.7; 1.7]	-0.1 [-0.8; 3.7]	1.8 [0.8; 4.7]	3.5 [-1.9; 117.4]	0.6 [0.3; 1.8]	5.7 [2.3; 43.7]	0.22	0.10
	MU	44.2 [17.5; 50.7]	-1.7 [-22.4; 26.4][a]	48.2 [12.0; 92.5]	60.0 [-4.8; 610.1][b]	17.5 [13.3; 52.7]	259.1 [30.3; 401.8][b]	**0.019**†	**0.005**†
	SAT	147.1 [83.2; 266.5]	-87.9 [-168.1; 1.7][a]	104.9 [65.9; 486.5]	101.0 [-68.3; 539.1][a,b]	112.7 [57.9; 158.8]	277.4 [12.2; 2915.9][b]	**0.017**†	**0.005**†
	Total	209.1 [107.5; 345.1]	-61.5 [-190.5; -0.0][a]	226.9 [98.0; 579.0]	541.9 [25.7; 2117.8][a,b]	140.6 [109.9; 188.0]	511.1 [201.0; 3188.3][b]	**0.006**†	**0.001**†
Cer	C16:0	152.3 [71.2; 199.0]	-23.3 [-125.4; 7.5][a]	174.9 [88.4; 234.0]	1319.0 [-2.6; 2105.1][b]	150.0 [107.1; 326.5]	1255.3 [481.8; 4006.2][b]	**0.0005**†	**0.0001**†
	C16:1	5.0 [2.9; 7.4]	0.3 [-4.3; 1.5][a]	5.5 [2.7; 7.2]	10.1 [-1.4; 24.6][a,b]	6.2 [3.4; 9.4]	17.9 [6.6; 45.5][b]	**0.004**†	**0.002**†
	C18:0	35.2 [17.6; 87.2]	-2.4 [-17.2; 17.2][a]	49.5 [22.8; 106.0]	592.0 [6.9; 782.4][b]	61.3 [29.3; 102.6]	249.0 [103.1; 1408.6][b]	**0.002**†	**0.0005**†
	C18:1	30.9 [18.2; 53.3]	-12.3 [-21.9; -1.6][a]	24.1 [17.6; 54.0]	224.5 [2.2; 315.6][b]	31.6 [25.7; 52.5]	282.3 [124.6; 786.7][b]	**0.0003**†	**0.0001**†
	C20:0	3.2 [2.3; 13.3]	-0.5 [-2.4; 3.1][a]	8.8 [5.3; 11.7]	78.5 [-0.9; 183.9][b]	12.1 [4.6; 14.5]	52.4 [18.2; 234.4][b]	**0.004**†	**0.001**†
	C20:1	1.6 [0.9; 3.8]	-0.4 [-2.4; 0.0][a]	2.2 [1.2; 4.2]	14.0 [0.6; 49.6][b]	3.1 [1.1; 5.8]	20.1 [6.1; 38.0][b]	**0.0005**†	**0.00009**†
	C22:0	20.6 [10.1; 41.6]	-2.6 [-19.1; 3.5][a]	26.1 [16.8; 61.9]	923.9 [27.2; 1675.7][b]	37.5 [18.1; 55.3]	740.8 [237.4; 3584.8][b]	**0.00001**†	**0.00001**†
	C22:1	1.8 [0.6; 3.4]	0.1 [-1.3; 0.6]	3.6 [2.0; 6.9]	31.3 [0.6; 60.2]	4.9 [2.6; 9.7]	23.4 [0.9; 120.9]	**0.032**†	**0.008**†
	C24:0	24.4 [12.5; 48.8]	-5.4 [-14.7; 3.0][a]	25.4 [16.0; 64.2]	685.8 [23.5; 1602.6][b]	41.4 [22.9; 53.6]	820.3 [174.1; 3408.2][b]	**0.00002**†	**0.00001**†
	C24:1	19.5 [9.3; 40.2]	-4.1 [-13.3; 4.1][a]	28.7 [17.0; 66.6]	90.0 [-13.7; 336.5][a,b]	47.4 [26.3; 76]	115.1 [26.1; 558.5][b]	**0.006**†	**0.002**†
	C26:0	3.3 [2.8; 4.7]	-0.5 [-2.0; 0.0][a]	3.2 [1.9; 4.8]	10.9 [-0.4; 39.5][a,b]	4.0 [2.2; 4.6]	18.4 [3.5; 69.9][b]	**0.006**†	**0.002**†
	C26:1	2.7 [1.4; 3.0]	-1.0 [-2.2; -0.2][a]	1.8 [1.0; 2.9]	1.2 [-1.4; 6.7][a,b]	1.8 [0.7; 3.5]	2.5 [-0.1; 15.4][b]	**0.035**†	**0.012**†
	MU	71.0 [38.7; 101.4]	-21.5 [-31.0; -7.2][a]	68.9 [48.7; 114.6]	401.2 [-26.5; 796.9][b]	105.0 [62.3; 162.1]	628.2 [173.9; 1384.2][b]	**0.003**†	**0.001**†
	SAT	245.9 [118.2; 397.9]	-17.6 [-184.7; -11.7][a]	342.8 [146.8; 422.7]	4302.4 [51.1; 6853.3][b]	317.0 [191.7; 539.0]	3123.8 [1030.4; 13955.2][b]	**0.0002**†	**0.00006**†
	Total	291.6 [145.5; 485.5]	-22.7 [-215.8; -19.8][a]	403.7 [195.5; 537.2]	4703.6 [15.4; 7924.2][b]	452.7 [263.3; 661.1]	3850.0 [1204.2; 15728.7][b]	**0.0002**†	**0.00006**†

Data are presented as median [q1; q3] (control n = 9; 3g-PL n = 7; 5g-PL n = 8) and expressed in nmol / g of lyophilized fecal samples. Results are presented based on the assumption of sphingosine d18:1 as the major sphingoid base. *P* values presented in bold highlight significant intervention effect. P_{group} represents *P* value associated with group effect as calculated by generalized linear model, while P_{PL} represents *P* value associated with binary effect of milk PL compared to control. † *P* value remains significant (< 0.05) after adjustment for clinical center, quartiles of volunteer age and waist circumference. [a,b] Different superscript letters indicate statistically different intervention effects between groups as calculated by *post hoc* analysis. Cer: ceramides; MU: monounsaturated species; PL: polar lipids; SAT: saturated species SM: sphingomyelin.

Supplemental Table 5. Daily self-reported food intake (VALOBAB-C trial).

All foods (including test cheese)	Control (n=18)		3g-PL (n=18)		5g-PL (n=20)		P_{group}
	V1	ΔV2-V1	V1	ΔV2-V1	V1	ΔV2-V1	
**Energy (kcal)**	1755.8 ±95.6	36.5 ±66.7	1725.7 ±70.4	-4.4 ±57.5	1999.0 ±77.2	-113.9 ±97.9	0.37
**Protein (% TEI)**	19.75 ±0.83	-0.24 ±0.72	18.54 ±0.64	-0.48 ±0.96	18.31 ±0.56	0.47 ±0.58	0.64
**Fat (% TEI)**	32.75 ±0.91	-0.60 ±0.93	32.57 ±1.02	-0.68 ±1.33	33.70 ±1.22	0.17 ±1.01	0.83
**Carbohydrates (% TEI)**	43.21 ±1.43	1.21 ±1.23	44.51 ±1.20	1.15 ±1.25	43.44 ±1.37	-0.59 ±1.02	0.45
**Simple sugars (g)**	68.18 ±5.02	4.62 ±3.90	68.60 ±6.20	4.81 ±5.02	80.45 ±5.30	1.43 ±4.58	0.83
**Fibers (g)**	17.61 ±1.14	-0.22 ±1.16	17.46 ±1.00	-1.20 ±0.65	19.43 ±1.12	-1.02 ±1.19	0.78
**Cholesterol (mg)**	271.67 ±25.93	15.01 ±24.24	288.53 ±34.84	20.37 ±43.92	322.61 ±31.60	87.54 ±34.84	0.27
**Alcohol (% TEI)**	0.63 ±0.32	-0.36 ±0.26	0.72 ±0.25	-0.19 ±0.31	1.18 ±0.38	-0.09 ±0.27	0.79
**SFAs (g)**	17.97 ±1.72	-0.09 ±1.29	19.03 ±1.90	-1.31 ±1.89	21.57 ±1.89	0.08 ±1.87	0.82
**MUFAs (g)**	18.53 ±2.02	-0.45 ±2.31	18.45 ±1.52	-0.97 ±1.70	22.49 ±1.97	-2.87 ±2.22	0.69
**PUFAs (g)**	7.06 ±0.63	0.17 ±0.88	6.29 ±0.44	0.52 ±0.86	9.11 ±0.70	-1.59 ±0.93	0.20

Intake was reported the 4 days before (V1) and after (V2) 4 weeks of consumption of 100 g of cream cheese / day with or without PL. Data are presented as mean ± SEM. P_{group} represents *P*-value associated with group effect as calculated by generalized linear model. *P* values after adjustment for clinical center, quartiles of volunteer age and waist circumference were also not significant (> 0.05). MUFA: monounsaturated fatty acid; PL: polar lipids; PUFA: polyunsaturated fatty acid; SFA: saturated fatty acid; TEI: total energy intake.

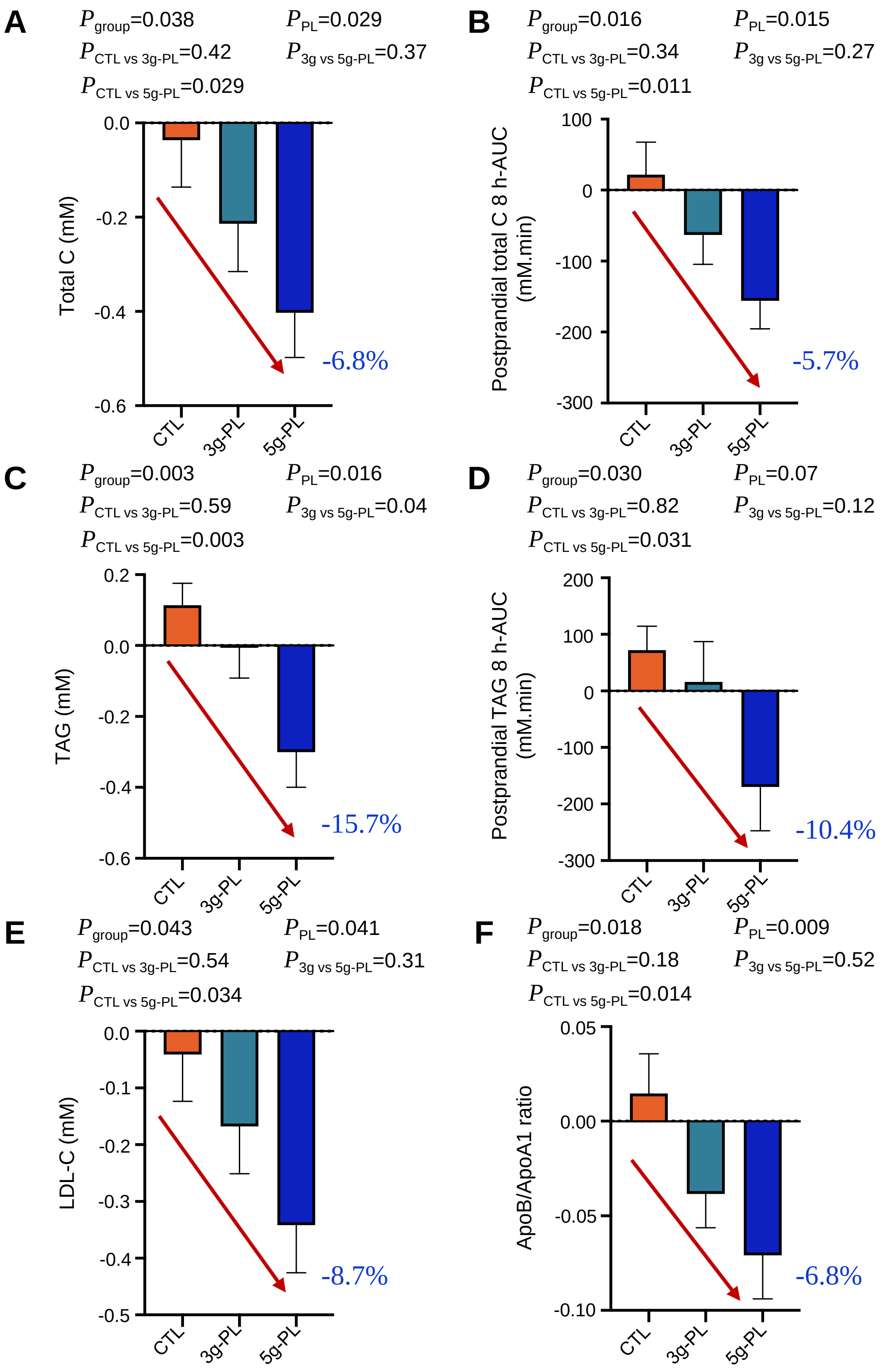

Supplemental Figure 1. Summary of the main VALOBAB-C trial outcomes (Vors *et al*. Gut 2020): milk SP *via* milk PL supplementation during 4 weeks reduces specific biomarkers of cardiovascular risk, at fasting and during the postprandial period. (**A**) fasting serum total C. (**B**) 8h-postprandial AUC of serum total C. (**C**) fasting serum TAG. (**D**) 8 h-postprandial AUC of serum TAG. (**E**) fasting serum LDL-C. (**F**) fasting plasma ApoB/ApoA1 ratio. Data are presented as mean ± SEM (n = 19 for control and 3g-PL groups, n = 20 for 5g-PL group). Statistical analysis was done using linear mixed model followed by Tukey-Kramer's *post hoc* test (P_{group} and $P_{posthoc}$) and P_{PL} represents P value associated with binary effect of milk PL compared to control. $P_{posthoc}$ corresponds altogether to $P_{CTL\ vs\ 3g\text{-}PL}$, $P_{CTL\ vs\ 5g\text{-}PL}$ and $P_{3g\ vs\ 5g\text{-}PL}$. Apo: apolipoprotein; C: cholesterol; CTL: control; LDL: low density lipoprotein; PL: polar lipids; TAG: triacylglycerols.

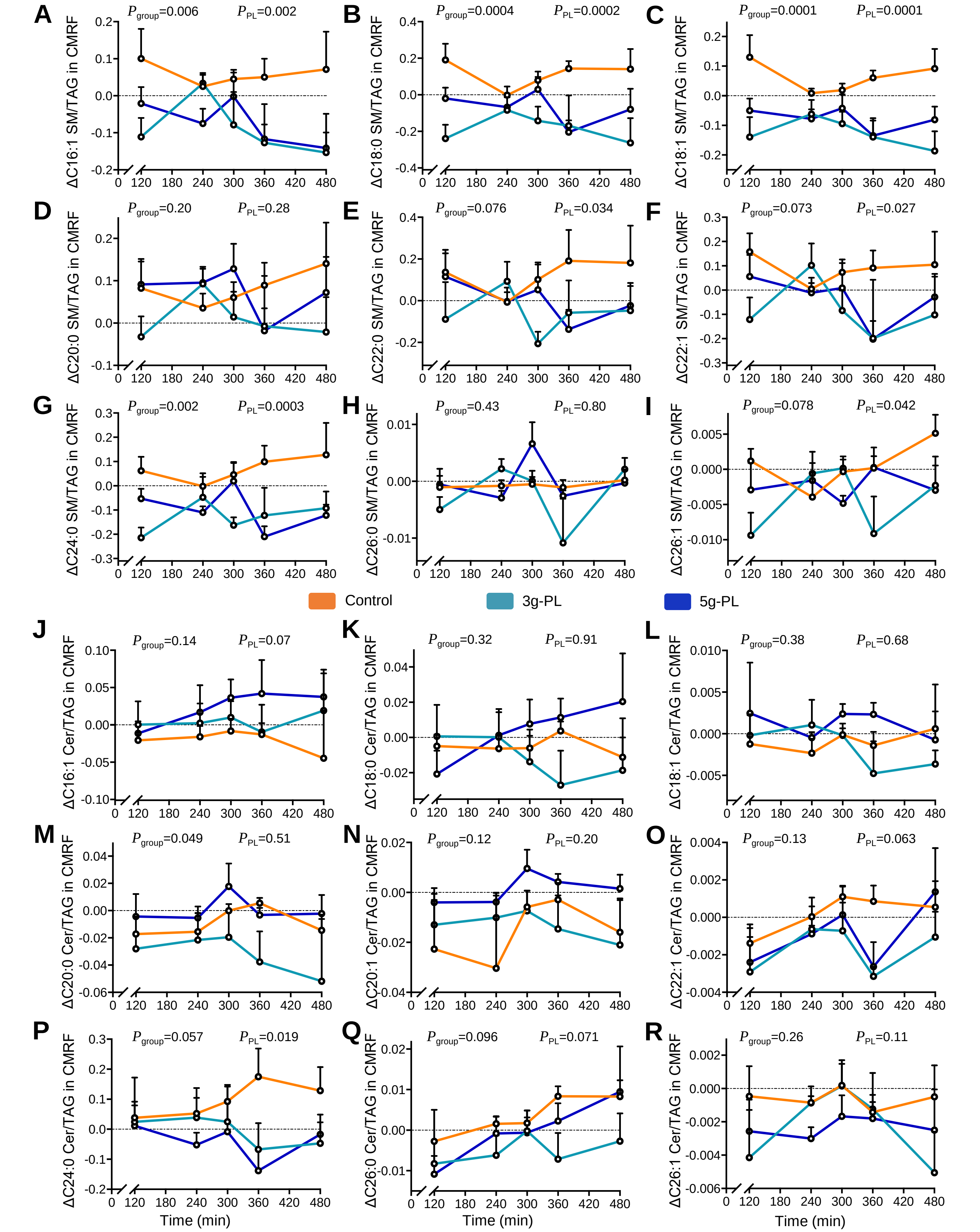

Supplemental Figure 2. Milk PL modulate SM and Cer molecular composition of plasma CMRF (complementary to Figure 3 and Supplemental Table 3, VALOBAB-C trial). Data are presented as mean ± SEM (n = 6 / group) and correspond to ΔSM (**A-I**) and ΔCer (**J-R**) species of interest in CMRF. Data are expressed in µmol / mmol of plasma CMRF TAG. Cer, ceramides; CMRF: chylomicron-rich fractions; CTL: control; PL: polar lipids; SM: sphingomyelins; TAG: triacylglycerols.

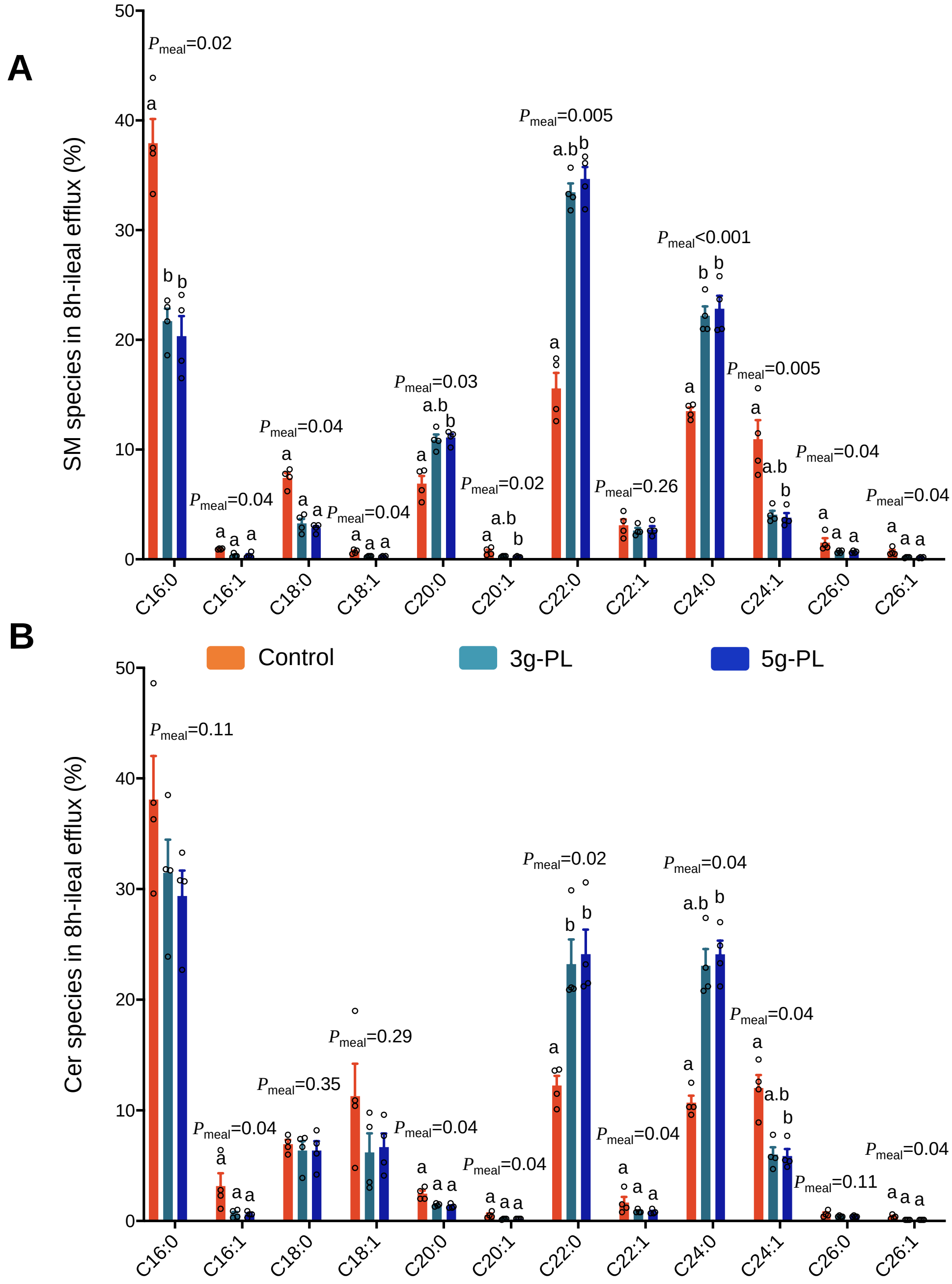

Supplemental Figure 3. Milk SP *via* milk PL ingestion increase SM and Cer molecular profiles in 8h ileal efflux (complementary to Figure 4, VALOBAB-D trial). Cumulated enrichment over 0-480 min of SM (**A**) and Cer (**B**) species. Data are presented as mean ± SEM and expressed as percentage of each SM or Cer species in total analyzed SM or Cer, respectively (n = 4). Results are presented based on the assumption of sphingosine d18:1 as the major sphingoid base. Empty circles correspond to individual values. Statistical analysis was done using Friedman's test followed by Dunn's *post hoc* test (non-normal data) or RM one-way ANOVA followed by a Tukey's *post hoc* test (normal data). [a.b] Different letters indicate statistically different intervention effects between groups ($P < 0.05$) as calculated by *post hoc* analysis. Cer: ceramides; CTL: control; PL: polar lipids; SP: sphingolipids; SM: sphingomyelin.

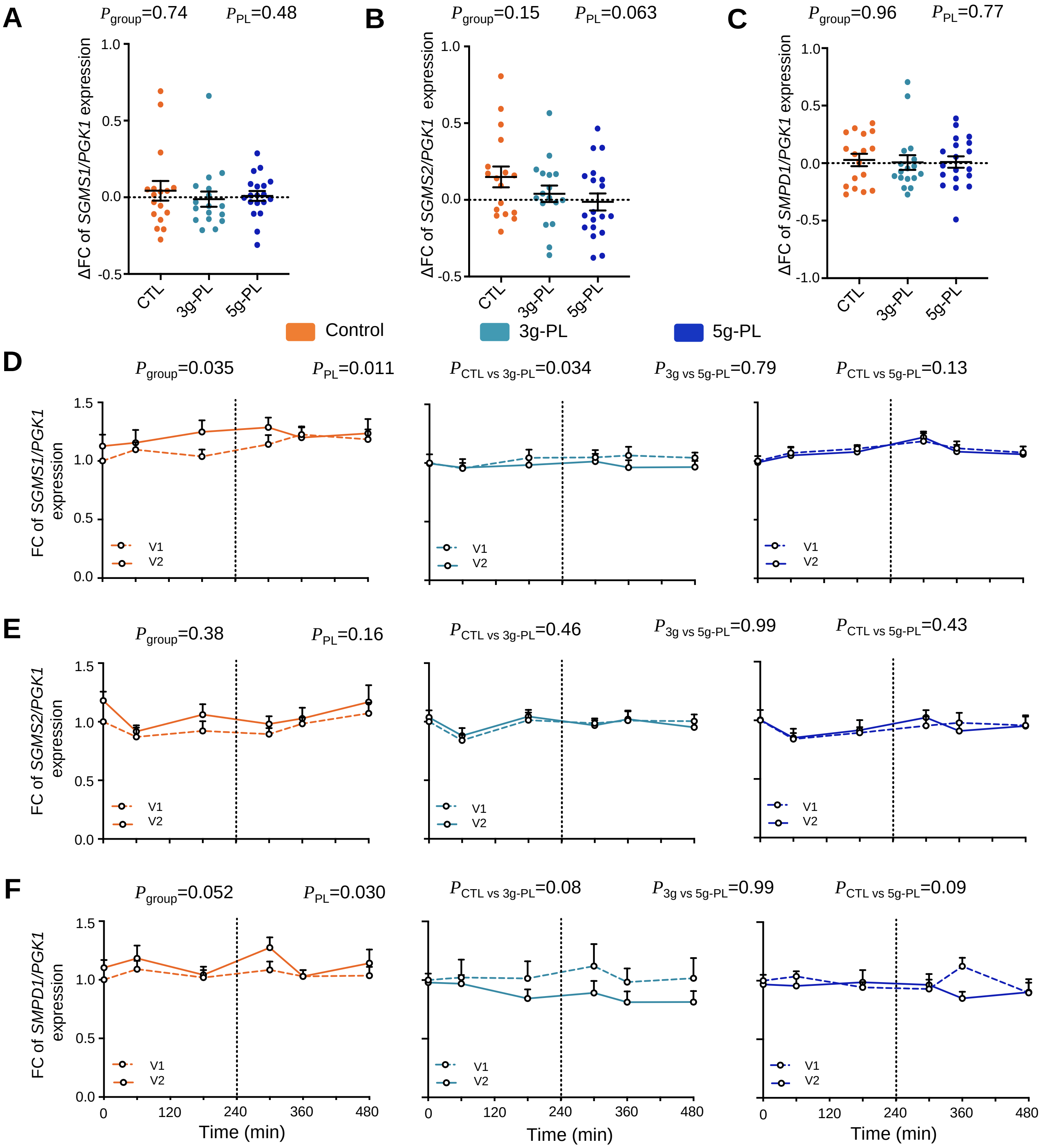

Supplemental Figure 4. Impact of 4-week milk SP *via* milk PL supplementation on gene expression in whole blood cells of key enzymes involved in SP metabolism (VALOBAB-C trial). (**A**), (**B**), (**C**) intervention impact on the gene expression of *SGMS1*, *SGMS2* and *SMPD1* in whole blood cells at fasting. (**D**), (**E**), (**F**) gene expression of these genes along the 8 h postprandial period before (V1: dotted line) and after (V2: full line) the daily consumption of 100 g of cheese with or without PL during 4 weeks. Gene expressions are normalized by the housekeeping gene *PGK1*. Data are presented as mean ± SEM (n = 17 for control and 3g-PL groups, n = 19 for 5g-PL group in panels **A-C**. n = 10 / group in panels **D-F**). Statistical analysis was done using a linear mixed model followed by Tukey-Kramer's *post hoc* test (P_{group} and $P_{posthoc}$) and P_{PL} represents *P* value associated with binary effect of milk PL compared to control. $P_{posthoc}$ corresponds altogether to $P_{CTL\ vs\ 3g\text{-}PL}$; $P_{CTL\ vs\ 5g\text{-}PL}$ and $P_{3g\ vs\ 5g\text{-}PL}$. CTL: control; FC: fold-change *versus* fasting gene expression at V1; PGK1: phosphoglycerate kinase 1; PL: polar lipids; SGMS1/2: sphingomyelin synthase 1 and 2; SMPD1: sphingomyelin phosphodiesterase 1.

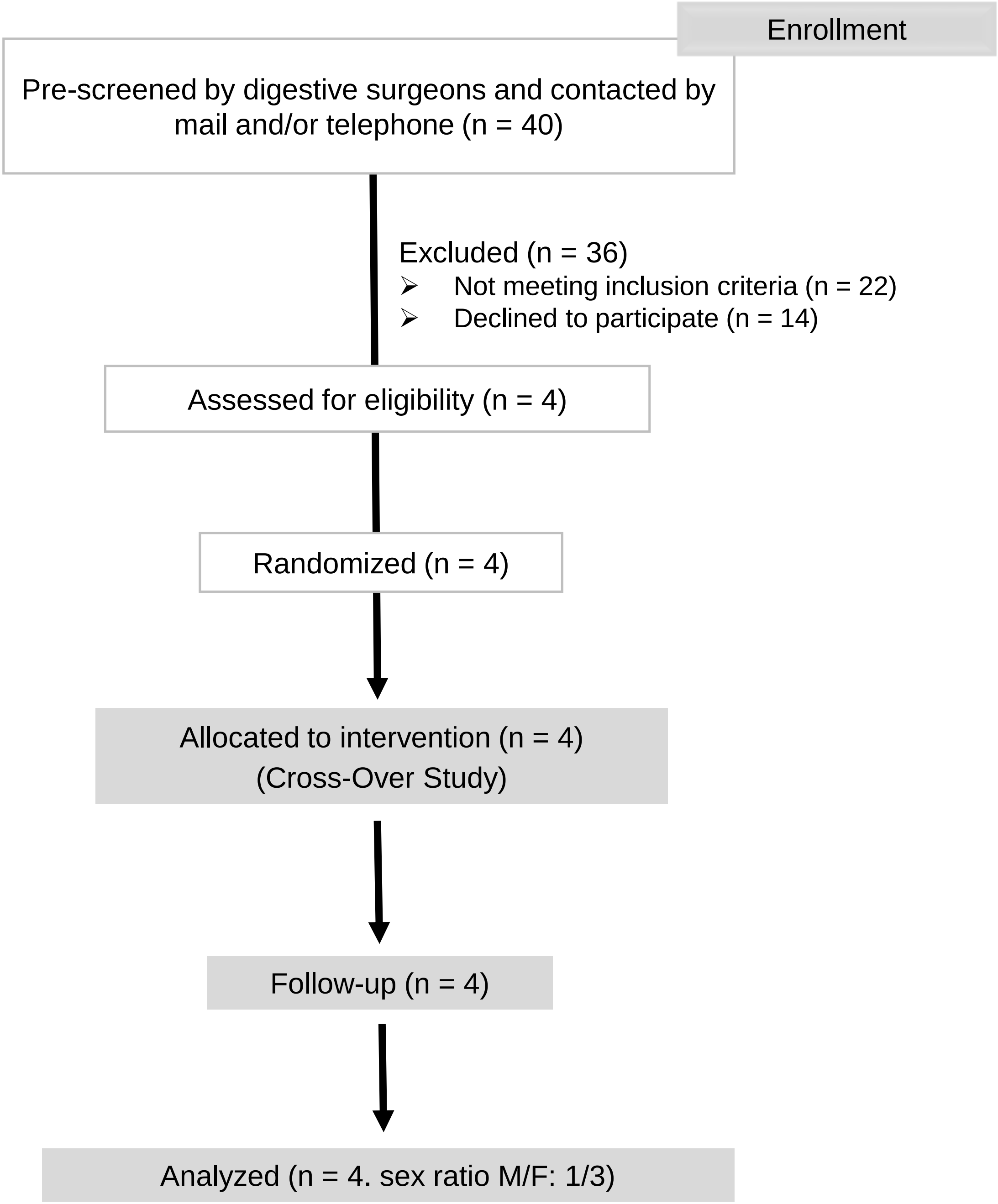

Supplemental Figure 5. Flow chart diagram of the VALOBAB-D clinical trial.
